# EEF2-inactivating toxins engage the NLRP1 inflammasome and promote epithelial barrier disruption

**DOI:** 10.1084/jem.20230104

**Published:** 2023-08-29

**Authors:** Miriam Pinilla, Raoul Mazars, Romain Vergé, Leana Gorse, Margaux Paradis, Bastien Suire, Karin Santoni, Kim Samirah Robinson, Gee Ann Toh, Laure Prouvensier, Stephen Adonai Leon-Icaza, Audrey Hessel, David Péricat, Marlène Murris, Hélène Guet-Revillet, Anthony Henras, Julien Buyck, Emmanuel Ravet, Franklin L. Zhong, Céline Cougoule, Rémi Planès, Etienne Meunier

**Affiliations:** 1 Institute of Pharmacology and Structural Biology, University of Toulouse, CNRS, Toulouse, France; 2 Lee Kong Chian School of Medicine, Nanyang Technological University, Singapore, Singapore; 3 Skin Research Institute of Singapore, Singapore, Singapore; 4 UFR Medicine and Pharmacy, INSERM U1070, University of Poitiers, Poitiers, France; 5 Department of Pneumology, Hospital Larrey, Toulouse, France; 6 University Hospital of Toulouse, Toulouse, France; 7 Center of Integrative Biology, University of Toulouse, CNRS, Toulouse, France; 8 Invivogen, Toulouse, France

## Abstract

Human airway and corneal epithelial cells, which are critically altered during chronic infections mediated by *Pseudomonas aeruginosa*, specifically express the inflammasome sensor NLRP1. Here, together with a companion study, we report that the NLRP1 inflammasome detects exotoxin A (EXOA), a ribotoxin released by *P. aeruginosa* type 2 secretion system (T2SS), during chronic infection. Mechanistically, EXOA-driven eukaryotic elongation factor 2 (EEF2) ribosylation and covalent inactivation promote ribotoxic stress and subsequent NLRP1 inflammasome activation, a process shared with other EEF2-inactivating toxins, diphtheria toxin and cholix toxin. Biochemically, irreversible EEF2 inactivation triggers ribosome stress–associated kinases ZAKα- and P38-dependent NLRP1 phosphorylation and subsequent proteasome-driven functional degradation. Finally, cystic fibrosis cells from patients exhibit exacerbated P38 activity and hypersensitivity to EXOA-induced ribotoxic stress–dependent NLRP1 inflammasome activation, a process inhibited by the use of ZAKα inhibitors. Altogether, our results show the importance of *P. aeruginosa* virulence factor EXOA at promoting NLRP1-dependent epithelial damage and identify ZAKα as a critical sensor of virulence-inactivated EEF2.

## Teaser

Here, Pinilla and colleagues address the ability of the human NLRP1 inflammasome-forming sensor to detect translation inactivation by bacterial toxins in healthy and cystic fibrosis airway and corneal epithelial cells.

## Graphical Abstract

**Figure:**
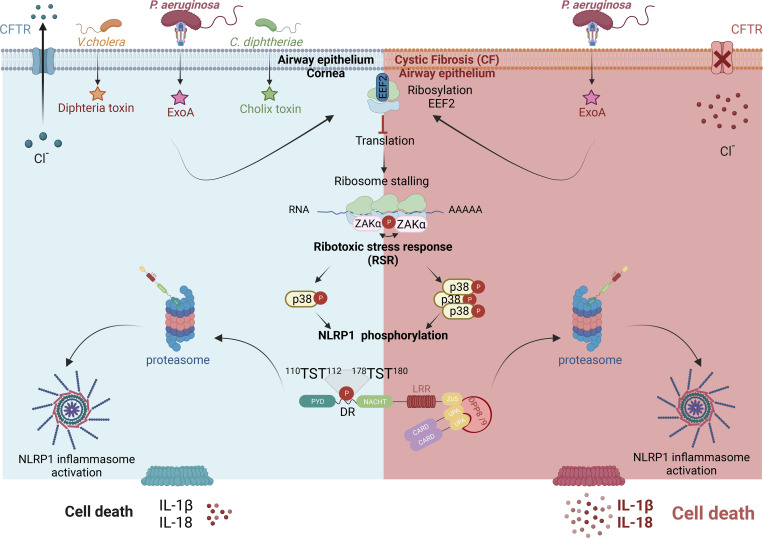

## Introduction

*Pseudomonas aeruginosa* is an opportunistic bacterial pathogen that can cause acute and chronic life-threatening infections (Qin et al., 2022; Maurice et al., 2018). Due to widespread antibiotic resistance and its adaptation to the airway, skin, and cornea of immune-compromised patients (e.g., ciliated dyskinesia, chronic granulomatous diseases, cystic fibrosis [CF]), *P. aeruginosa* is listed as an important ESKAPE pathogen in the World Health Organization list of microbes of concerns (De Oliveira et al., 2020). *P. aeruginosa* can trigger acute infections, thanks to the expression of a type 3 secretion system (T3SS), which leads to a robust inflammatory reaction mostly mediated by monocytes/macrophages and neutrophils (Qin et al., 2022). However, during chronic infections, *P. aeruginosa* switches into a metabolically different state that represses T3SS expression and allows the expression and secretion of a different arsenal of effectors involved in the formation/maintenance of biofilm-like structures, which are extremely resistant to antibiotic treatments (Qin et al., 2022). In addition, the secretion of numerous matrix-remodeling factors such as (phospho)lipases, proteases, siderophores, and oxidative and toxic molecules strongly contribute to immune response deviation as well as to tissue damages, including epithelial barrier disruption (Qin et al., 2022). In this context, inflammatory mediator analysis from CF patients chronically infected with *P. aeruginosa* highlights an enrichment in inflammasome-derived cytokine IL-1β, suggesting that during the chronic step of *P. aeruginosa* infection one or many inflammasomes might be activated (Bonfield et al., 2012). Given the prominent epithelial cell damage observed in *P. aeruginosa* infected patients and the poorly addressed function of the epithelial barrier in antibacterial defense, we hypothesized that some epithelial inflammasomes might respond to one or various factors released by *P. aeruginosa* during chronic infections.

Inflammasomes, which mostly belong to the nucleotide-binding domain leucine-rich repeats (NLRs) and AIM2-like receptor families, are a subset of germline-encoded innate immune sensors that detect and respond to various signs of infections and environmental stresses (Lacey and Miao, 2020). Upon activation, inflammasome-forming sensors assemble a cytosolic supramolecular structure composed of the sensor/receptor, the adaptor protein apoptosis-associated speck-like protein containing a CARD (ASC), and the protease Caspase-1. Inflammasome assembly leads to Caspase-1–dependent maturation and release of inflammatory cytokines IL-1β and IL-18 as well as to pyroptosis, a proinflammatory form of cell death characterized by gasdermin (D)-driven pore formation and Ninjurin-1–dependent membrane disassembly (Lacey and Miao, 2020; Broz and Dixit, 2016; Kayagaki et al., 2021, 2023; Borges et al., 2022; Degen et al., 2023; Bjanes et al., 2021).

Specifically, human NLRP1 is notable among other inflammasome sensors because of its expression in, but not restricted to, various epithelia (e.g., keratinocytes, airways, and cornea). In addition, rare germline mutations and single-nucleotide polymorphisms in NLRP1 are associated with infection sensitivity, skin, corneal, and intestinal inflammatory disorders as well as with asthma susceptibility in humans, hence underlying an important function of NLRP1 at triggering an innate immune response in various epithelia (Zhong et al., 2016; Griswold et al., 2022).

A conserved mechanism of NLRP1 inflammasome activation relies on proteasome-driven degradation of the NLRP1 N-terminal autoinhibitory fragment (NT) and the subsequent oligomerization of the released C-terminal fragment (CT), which nucleates the NLRP1 inflammasome assembly. In this regard, recent studies identified NLRP1 as a cell sensor of redox and proteotoxic stressors (Wang et al., 2023; Orth-He et al., 2023). Further studies also unveiled human-specific ligands/pathways driving NLRP1 inflammasome activation, including the viral double-stranded RNA, 3C, and 3CL proteases from rhinovirus and SARS-CoV-2 viruses as well as host ZAKα and P38 stress kinase-driven phosphorylation upon exposure to ribosome stressors (UV-B irradiation, anisomycin; Tsu et al., 2021; Robinson et al., 2020, 2022; Griswold et al., 2022; Bauernfried et al., 2021; Jenster et al., 2023; Fenini et al., 2018; Planès et al., 2022). Although all those ligands/signaling pathways converge to human (h)NLRP1 inflammasome activation, the mechanisms involved still remain under investigation.

In this study, we discover that in human corneal and nasal epithelial cells, *P. aeruginosa* contributes to barrier disruption by secreting the eukaryotic elongation factor 2 (EEF2)-inactivating toxin exotoxin A (EXOA), which subsequently triggers assembly of the NLRP1 inflammasome and release of associated IL-1 cytokine as well as pyroptotic cell death. In this process, EXOA-inactivated EEF2 promotes ribotoxic stress response (RSR) and subsequent activation of the stress kinases ZAKα and P38 (Wu et al., 2020; Vind et al., 2020). Subsequently, activated ZAKα and P38 stimulate NLRP1 phosphorylation in its disordered region, functional degradation, and activation. Finally, CF cells from patients show exacerbated P38 activity and hypersensitivity to EXOA-induced ribotoxic stress-dependent NLRP1 inflammasome activation, a process reverted by the use of ZAKα inhibitors. Altogether, our results describe the ability of *P. aeruginosa* virulence factor EXOA at promoting NLRP1-dependent tissue damage and identify ZAKα as a critical sensor of bacterial pathogen-driven ribosome inactivation.

## Results and discussion

### *P. aeruginosa* infection activates the NLRP1 inflammasome in human corneal and airway epithelial cells

**Figure 1.**
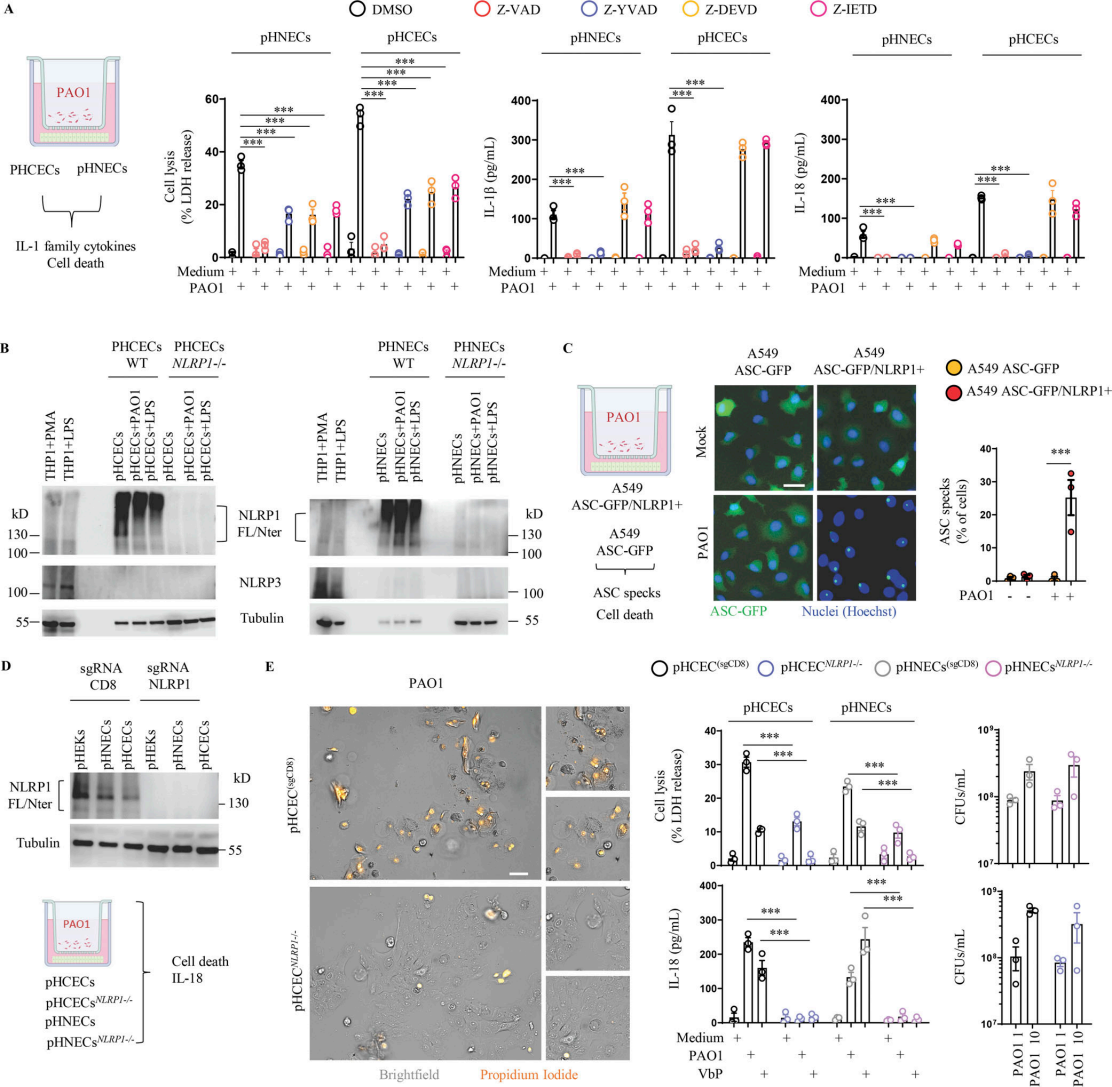
***P. aeruginosa* triggers human NLRP1 inflammasome activation in corneal and nasal epithelial cells. (A)** Cell lysis (LDH) and IL-1β/IL-18 release evaluation in pHCECs and pHNECs upon *P. aeruginosa* (PAO1, 1.10^5^ bacteria) co-culture for 24 h. When specified, the pan Caspase inhibitor (Z-VAD, 20 µM), Caspase-1 inhibitor (Z-YVAD, 20 µM), Caspase-3/7 inhibitor (Z-DEVD, 20 µM), and Caspase-8 inhibitor (Z-IETD, 20 µM) were used. ***P ≤ 0.001, two-way ANOVA with multiple comparisons. Values are expressed as mean ± SEM. Graphs show one experiment performed in triplicates at least three times. **(B)** Immunoblotting examination of NLRP1, NLRP3, and Tubulin in resting, PAO1-exposed as in A or LPS-primed pHCECs and pHNECs or in pHCECs and pHNECs genetically invalidated for *NLRP1* using CRISPR-Cas9. PMA (100 µg/ml)- or LPS (100 ng/ml)-primed THP1 monocytic cell line was used as a positive control for NLRP3 expression. Immunoblots show lysates from one experiment performed three times. **(C)** Florescence microscopy and associated quantifications of ASC-GFP specks in A549^NLRP1+/ASC-GFP^ and A549^NLRP1−/ASC-GFP^ reporter cell lines exposed to *P. aeruginosa* (PAO1, 1.10^5^ bacteria) for 24 h. ASC-GFP (green) pictures were taken in the dish after the infection. Images shown are from one experiment and are representative of *n* = 3 independent experiments; scale bars, 10 µm. ASC complex percentage was performed by determining the ratios of cells positive for ASC speckles on the total nuclei (Hoechst). At least 10 fields from each experiment were analyzed. Values are expressed as mean ± SEM. ***P ≤ 0.001, one-way ANOVA. **(D)** Immunoblotting characterization of genetic invalidation of *NLRP1* in pHCECs and pHNECs population using CRISPR-Cas9 and microscopy visualization of plasma membrane permeabilization (PI incorporation, orange) in pHCECs co-cultured with PAO1 (1.10^5^ bacteria) for 24 h. **(E)** sgRNA CD8 (SgCD8) was used as control and served as WT cells during subsequent experiments described in E. Images shown are from one experiment and are representative of *n* = 3 independent experiments; scale bars, 20 µm. Cell lysis (LDH), IL-18 release, and CFU evaluation in WT (SgCD8, D) or *NLRP1*-deficient pHCECs and pHNECs, upon VbP (15 µM) treatment or *P. aeruginosa* (PAO1, 1.10^5^ bacteria) co-culture for 24 h. For CFU analysis 1 × 10^4^ (MOI 1) or 1 × 10^5^ (MOI 10) bacteria were used. ***P ≤ 0.001, two-way ANOVA with multiple comparisons. Values are expressed as mean ± SEM. Graphs show one experiment performed in triplicates at least three times. Source data are available for this figure: SourceData F1.

**Figure S1.**
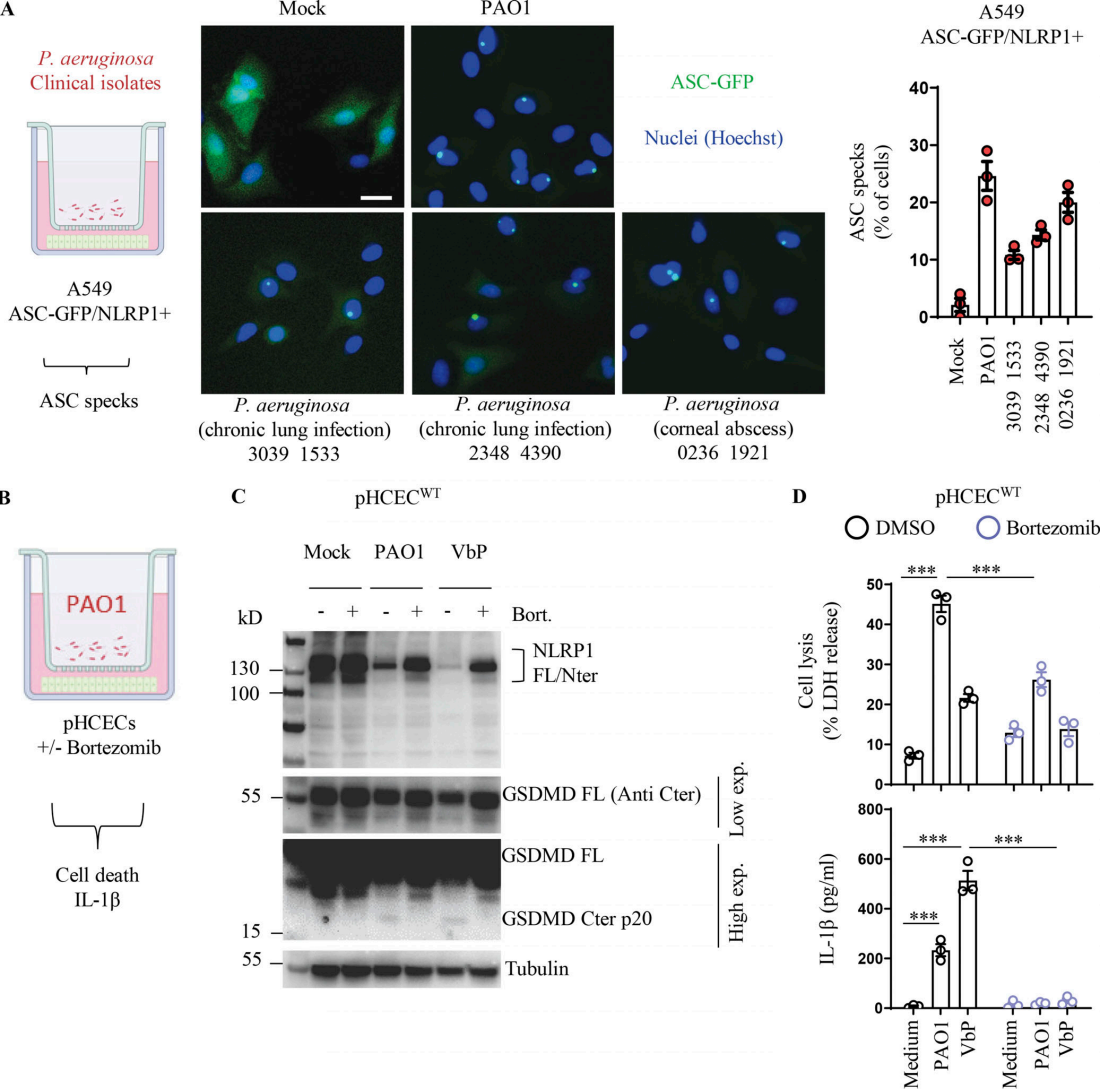
***P. aeruginosa*–activated hNLRP1 inflammasome requires proteasome activity. (A)** Fluorescence microscopy and associated quantifications of ASC-GFP specks in A549^NLRP1+/ASC-GFP^ reporter cell lines exposed to 1 × 10^5^ *P. aeruginosa* clinical isolates from patients with infected lung (strains 3039 1533 and 2348 4390) or with infected cornea (strain 0236 1921) for 24 h. ASC-GFP (green) pictures were taken in the dish after infection. Images shown are from one experiment and are representative of *n* = 3 independent experiments; scale bars, 10 µm. ASC complex percentage was performed by determining the ratios of cells positive for ASC speckles on the total nuclei (Hoechst). At least 10 fields from each experiment were analyzed. Values are expressed as mean ± SEM. One-way ANOVA. **(B)** Schematic drawing of *P. aeruginosa* co-culture experiments performed with human corneal epithelial cells. **(C)** Immunoblotting of NLRP1, Gasdermin-D, and Tubulin in pHCECs upon VbP (15 µM) treatment or *P. aeruginosa* (PAO1, 1.10^5^ bacteria) co-culture for 24 h in presence/absence of proteasome inhibitor bortezomib. Immunoblots show lysates from one experiment performed at least three times. **(D)** Cell lysis (LDH) and IL-1B release evaluation in pHCECs and pHNECs, upon VbP (15 µM) treatment or *P. aeruginosa* (PAO1, 1.10^5^ bacteria) co-culture for 24 h in presence/absence of proteasome inhibitor bortezomib. ***P ≤ 0.001, two-way ANOVA with multiple comparisons. Values are expressed as mean ± SEM from one experiment (in triplicate) performed at least three times. Source data are available for this figure: SourceData FS1.

As chronically infected patients exhibit both epithelial alterations and robust IL-1β cytokine levels, we wondered about the contribution of inflammasome response from epithelial compartments during *P. aeruginosa* infection. To mimic *P. aeruginosa* biofilm-like behavior, we relied on a transwell-adapted method that allowed bacteria to grow on top of a 0.4-µm porous membrane and where epithelial cells were seeded on the bottom of the wells (Fig. 1 A). In this context, we analyzed the inflammasome response (IL-1β/IL-18 and cell death) of primary human corneal (pHCECs) and nasal epithelial cells (pHNECs) co-cultured with *P. aeruginosa*. We observed that *P. aeruginosa* triggered robust cell death and IL-1β/IL-18 release both in pHNECs and pHCECs (Fig. 1 A). Importantly, the use of Z-YVAD, an inhibitor of Caspase-1 activity, underscored that IL-1β/IL-18 release fully depended on Caspase-1 activity while cell death was partly dependent on Caspase-1, Caspase-3, and Caspase-8 (Fig. 1 A). This suggests that the inflammasome in pHNECs and pHCECs could contribute to IL-1β/IL-18 and cell death in response to extracellular *P. aeruginosa*. To determine which inflammasome is involved, we immunoblotted for various inflammasome-forming sensors in those epithelial cells (Fig. 1 B). Although we failed at detecting NLRP3 expression in LPS-primed, PAO1-exposed, or resting cells, we could detect expression of the NLRP1 sensor both in nasal and corneal epithelial cells (Fig. 1 B; Robinson et al., 2020; Griswold et al., 2022). Hence, we hypothesized that NLRP1 might be a sensor of extracellular *P. aeruginosa* in the airway and corneal compartments. To address this question, we used our previously described epithelial lung A549-ASC-GFP reporter cell lines in which hASC-GFP and hNLRP1 constructs were stably introduced (Planès et al., 2022). Florescence microscopy and quantification of active inflammasome complexes (ASC-GFP^+^ puncta/Specks) in co-cultured reporter cells unveiled that *P. aeruginosa* exposure promoted the formation of inflammasome complexes as well as induced significant cell death specifically in NLRP1-expressing cells (Fig. 1 C). Importantly, the formation of inflammasome complexes also occurred in NLRP1-expressing cells exposed to three clinical isolates of *P. aeruginosa* (Fig. S1 A). To further determine the involvement of NLRP1 in primary cells at responding to extracellular *P. aeruginosa*, we removed NLRP1 expression in nasal and corneal epithelial cells by using CRISPR-Cas9 method (Fig. 1, B and D). Co-culture of WT and NLRP1-deficient cells with *P. aeruginosa* showed that *NLRP1*^−/−^ cells exhibited a defect in cell death (measured by propidium iodide [PI] incorporation and lactate dehydrogenase [LDH] release) as well as at releasing IL-1β/IL-18 cytokines (Fig. 1, D and E). As hNLRP1 inflammasome activation requires ubiquitination and subsequent proteasome-driven functional degradation, which releases the active NLRP1 C-Ter fragment, we next incubated pHCECs with PAO1 or with Val-boro-Pro (VbP), a known chemical activator of the NLRP1 inflammasome, in presence or absence of the proteasome inhibitor bortezomib (Fig. S1, B–D). Measure of the pyroptosis pore-forming protein Gasdermin-D processing, hNLRP1 degradation, cell death, and IL-1β release in pHCECs showed that proteasome inhibition strongly impaired those processes, hence suggesting that the hNLRP1 inflammasome activation by *P. aeruginosa* occurs in a proteasome-dependent manner in corneal and airway epithelial cells (Fig. S1, B–D).

Finally, to determine if hNLRP1-driven response could influence the extracellular growth of *P. aeruginosa*, we performed colony-forming unit (CFUs) quantification in primary WT and NLRP1-deficient nasal and corneal epithelial cells. After 24 h, we observed similar CFUs between WT and NLRP1-deficient cells co-cultured with PAO1, hence suggesting that NLRP1 activation does not directly influence extracellular PAO1 growth (Fig. 1 E).

### EEF2-inactivating EXOA promotes NLRP1 inflammasome response

**Figure 2.**
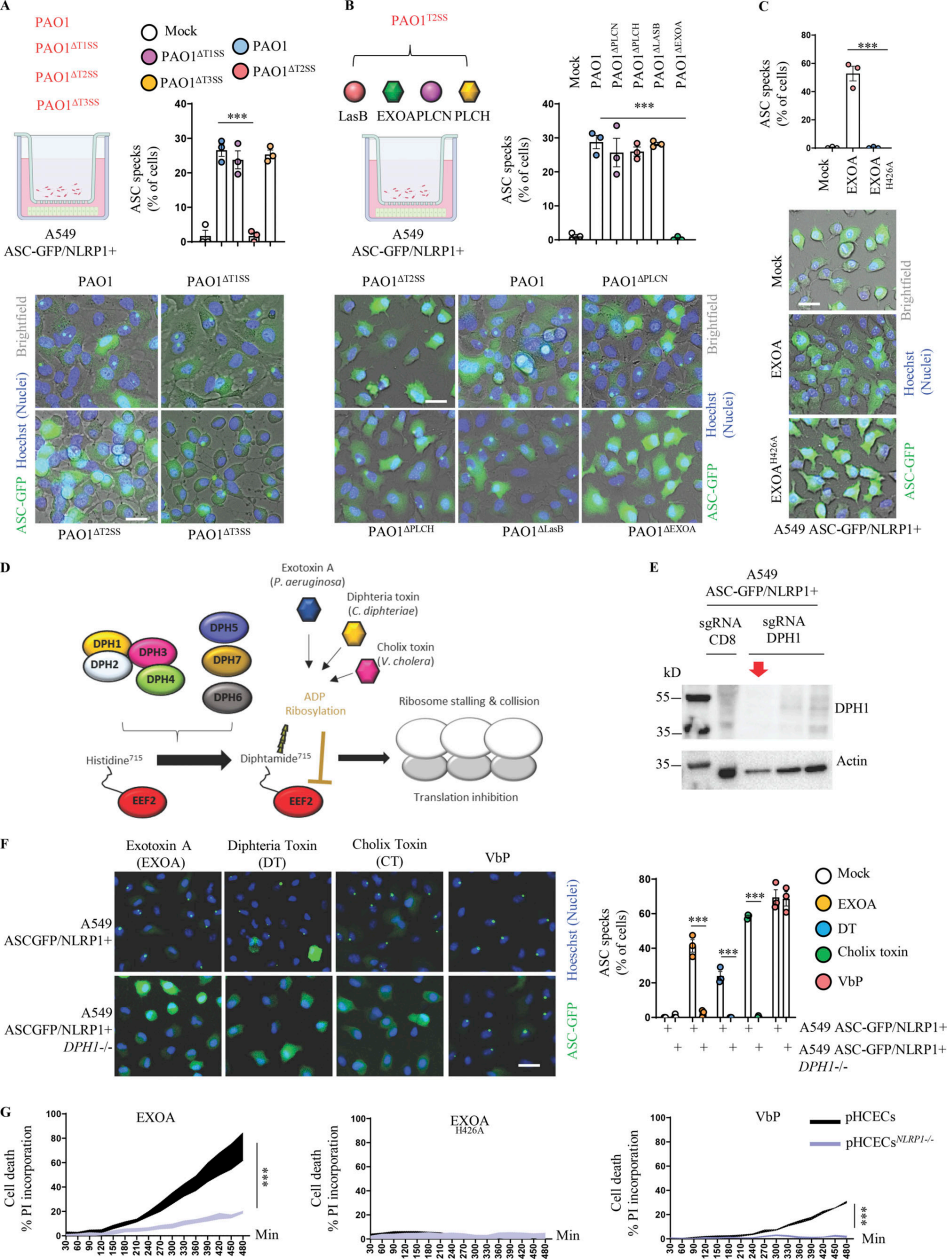
***P. aeruginosa* EEF2-inactivating EXOA promotes NLRP1 inflammasome response. (A)** Florescence microscopy and associated quantifications of ASC-GFP specks in A549^NLRP1+/ASC-GFP^ reporter cell lines exposed to 1 × 10^5^ *P. aeruginosa* (PAO1) and associated isogenic mutants for various secretion systems (PAO1^ΔT3SS^, PAO1^ΔT2SS^, PAO1^ΔT1SS^) for 24 h. ASC-GFP (green) pictures were taken in the dish after infection. Images shown are from one experiment and are representative of *n* = 3 independent experiments; scale bars, 10 µm. ASC complex percentage was performed by determining the ratios of cells positive for ASC speckles on the total nuclei (Hoechst). At least 10 fields from *n* = 3 independent experiments were analyzed. Values are expressed as mean ± SEM. ***P ≤ 0.001, one-way ANOVA. **(B)** Florescence microscopy and associated quantifications of ASC-GFP specks in A549^NLRP1+/ASC-GFP^ reporter cell lines exposed to 1 × 10^5^ *P. aeruginosa* (PAO1) and associated isogenic mutants for various T2SS virulence effectors (PAO1^ΔPLCN^, PAO1^ΔPLCH^, PAO1^ΔLASB^, and PAO1^ΔEXOA^) for 24 h. ASC-GFP (green) pictures were taken in the dish after infection. Images shown are from one experiment and are representative of *n* = 3 independent experiments; scale bars, 10 µm. ASC complex percentage was performed by determining the ratios of cells positive for ASC speckles on the total nulcei (Hoechst). At least 10 fields from *n* = 3 independent experiments were analyzed. Values are expressed as mean ± SEM. ***P ≤ 0.001, one-way ANOVA. **(C)** Florescence microscopy and associated quantifications of ASC-GFP specks in A549^NLRP1+/ASC-GFP^ reporter cell lines exposed to EXOA (10 ng/ml) or its catalytically dead mutant EXOA^H426A^ (500 ng/ml) for 10 h. ASC-GFP (green) pictures were taken in the dish after toxin exposure. Images shown are from one experiment and are representative of *n* = 3 independent experiments; scale bars, 10 µm. ASC complex percentage was performed by determining the ratios of cells positive for ASC speckles (green, GFP) on the total nuclei (Hoechst). At least 10 fields from *n* = 3 independent experiments were analyzed. Values are expressed as mean ± SEM. ***P ≤ 0.001, one-way ANOVA. **(D)** Schematic mechanism of *P. aeruginosa* EXOA and related toxins at mediating EEF2 ribosylation and inactivation and subsequent ribosome inactivation. **(E)** Immunoblotting characterization of genetic invalidation of *DPH1* in A549^NLRP1+/ASC-GFP^ cells using CRISPR-Cas9. The red arrow shows the selected KO cells for subsequent experiments. **(F)** Fluorescence microscopy and associated quantifications of ASC-GFP specks in A549^NLRP1+/ASC-GFP^ and A549^NLRP1+/ASC-GFP/*DPH1−*^ reporter cell lines exposed to VbP (15 µM), EXOA (10 ng/ml), cholix toxin (CT, 10 ng/ml), and diphtheria toxin (DT, 20 ng/ml) for 10 h. ASC-GFP (green) pictures were taken in the dish after toxin exposure. Images shown are from one experiment and are representative of *n* = 3 independent experiments; scale bars, 10 µm. ASC complex percentage was performed by determining the ratios of cells positive for ASC speckles (green, GFP) on the total nuclei (Hoechst). At least 10 fields from *n* = 3 independent experiments were analyzed. Values are expressed as mean ± SEM. ***P ≤ 0.001, one-way ANOVA. **(G)** Plasma membrane permeabilization determination over time using PI incorporation in WT or *NLRP1*-deficient pHCECs exposed to VbP (15 µM), EXOA (10 ng/ml) or EXOA^H426A^ (10 ng/ml) for indicated times. ***P ≤ 0.001, *T* test. Values are expressed as mean ± SEM from one experiment (in triplicate) performed at least three times. Source data are available for this figure: SourceData F2.

As the NLRP1 inflammasome responds to a yet-to-be-determined secreted product of *P. aeruginosa*, we next wondered about the identity of this factor. *P. aeruginosa* expresses various secretion systems (T1SS to T6SS) that allow either secreting or injecting various elements (Filloux, 2011, 2022). Using co-cultures of A549-ASC-GFP/NLRP1 reporter cell lines with PAO1 transposon mutants for different secretion systems, we observed that Type-2 Secretion System (T2SS)-deficient PAO1 specifically failed at inducing NLRP1 inflammasome complex assembly (Fig. 2 A). Among other factors released by PAO1 T2SS are phospholipases (PLCN, PLCH), elastase protease LASB, and the EXOA (Liao et al., 2022). Using PAO1 transposon mutants for each of those factors, we observed that only the PAO1 strain that is deficient for EXOA lost the ability of promoting the formation of the NLRP1 inflammasome in our reporter cell lines (Fig. 2 B). This suggests that T2SS-secreted EXOA is the major factor by which extracellular *P. aeruginosa* activates the NLRP1 inflammasome in epithelial cells.

**Figure S2.**
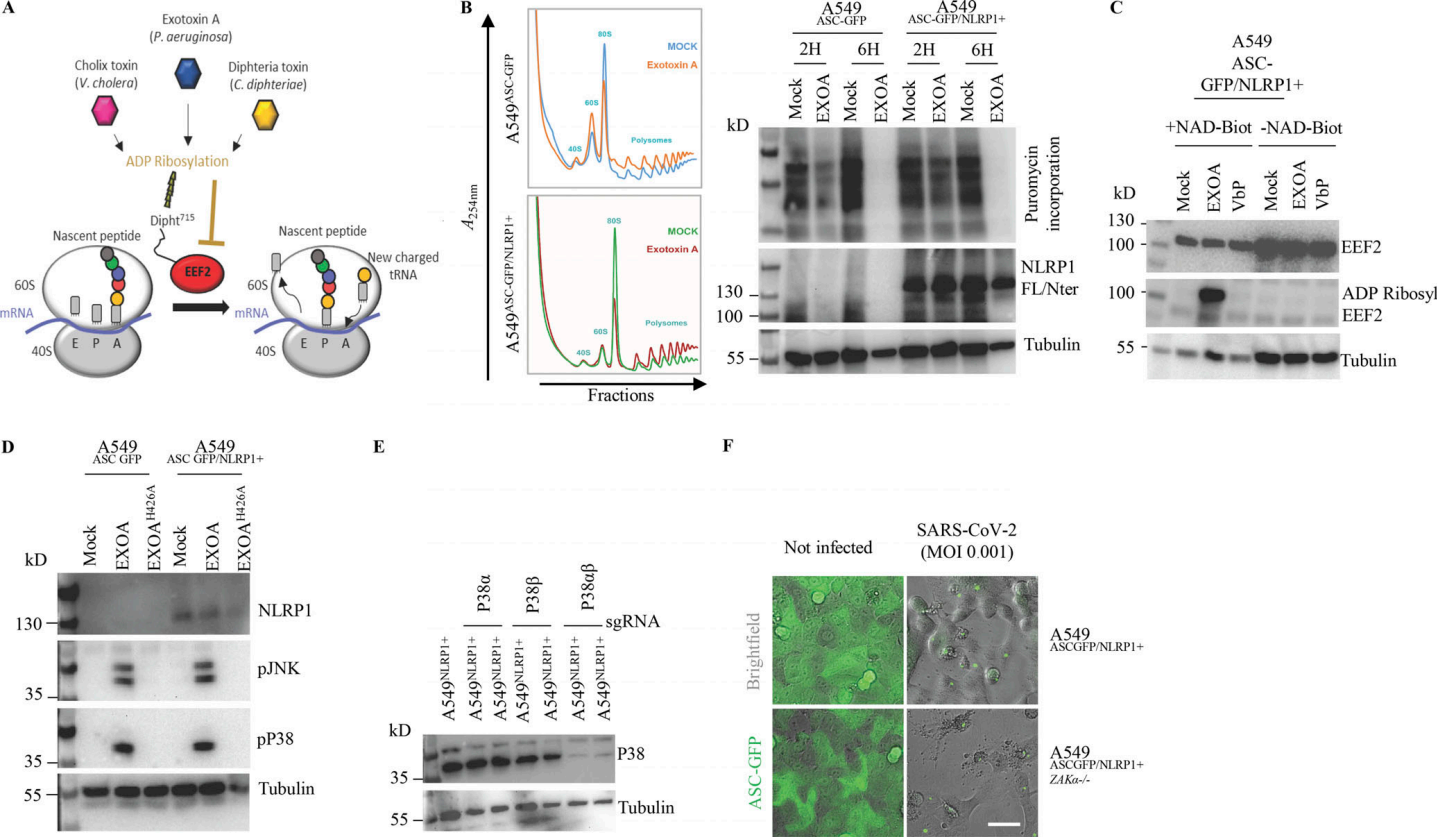
**Multiple EEF2-targeting toxins activate the hNLRP1 inflammasome in a ZAKα-dependent manner. (A)** Schematic mechanism of EXOA and related toxin-mediated translation inhibition. tRNA, transfer RNA; E, exit; P, peptidyl; A, aminoacyl. **(B)** Determination of ribosome inactivation in A549^NLRP1+/ASC-GFP^ and A549^NLRP1−/ASC-GFP^ reporter cell lines exposed to EXOA (10 ng/ml) for 2 and 6 h by measuring ribosome polysome accumulation and puromycin incorporation. Images are representative of one experiment performed at least three times. **(C)** Immunoblotting of ADP-ribosylated proteins, EEF2, and Tubulin in A549^NLRP1+/ASC-GFP^ cell lysates treated or not with VbP (15 µM) or EXOA (10 ng/ml) in the presence of Nicotinamide adenine dinucleotide-Biotin (NAD-Biot). Immunoblots show lysates from one experiment performed at least three times. **(D)** Immunoblotting of NLRP1, Tubulin, and phosphorylated P38 and JNK in A549^NLRP1+^ and A549^NLRP1−^ reporter cell lines exposed or not to EXOA (10 ng/ml) or its inactive mutant EXOA^H426A^ for 3 h. Immunoblots show lysates from one experiment performed at least three times. **(E)** Immunoblotting characterization of genetic invalidation of *P38α* and *P38β* in A549^NLRP1+/ASC-GFP^ cells using CRISPR-Cas9. Immunoblots show lysates from one experiment performed at least three times. **(F)** Fluorescence microscopy of ASC-GFP specks in A549^NLRP1+/ASC-GFP^ and A549^NLRP1+/ASC-GFP/*ZAKα*-^ reporter cell lines expressing hACE2 infected for 24 h with various SARS-CoV-2 MOI. ASC-GFP (green) pictures were taken in the dish after viral infection. Images shown are from one experiment and are representative of *n* = 3 independent experiments; scale bars, 10 µm. Source data are available for this figure: SourceData FS2.

EXOA is a ribotoxin that irreversibly ribosylates the elongation factor EEF2, which leads to ribosome inactivation (Jørgensen et al., 2005; Armstrong and Merrill, 2004), a process we also observed by determining ribosome polysomes accumulation (marker of alterations in translation machinery) and translation inhibition (puromycin incorporation as a marker of translation efficiency; Fig. S2, A and B). In this context, to determine if EXOA ribosylating activity was required for NLRP1 inflammasome response, we exposed reporter cells to recombinant EXOA and the catalytically dead mutant EXOA^H426A^, which is unable to promote EEF2 ribosylation (Roberts and Merrill, 2002; Fig. 2 C, and Fig. S2 C). Microscopy observation and quantifications of NLRP1 inflammasome complexes in reporter cells showed that EXOA but not its mutant triggered robust inflammasome formation (Fig. 2 C and Fig. S2 C). EEF2 ribosylation by EXOA requires the presence of a specific modified form of histidine 715 on human EEF2, namely diphthamide (Liu et al., 2004; Ivankovic et al., 2006; Fig. 2 D). This unique amino acid arises from the enzymatic modification of histidine by the so-called diphthamide enzymes (DPHs; Liu et al., 2004; Ivankovic et al., 2006). Hence, using our reporter cells, we genetically deleted *DPH1*, which is critical to initiate diphthamide synthesis (Fig. 2 E). Analysis of NLRP1 inflammasome complexes in response to EXOA showed that *DPH1*-deficient cells failed to assemble an active NLRP1 inflammasome complex (Fig. 2 F). As a control, activation of the NLRP1 infammasome by the Val-boro-Pro (VbP) molecule was not affected by the removal of *DPH1*, suggesting that EXOA specifically activates the NLRP1 inflammasome by promoting EEF2-ribosylation on the diphthamide 715 amino acid (Fig. 2 F). In addition to EXOA, two other bacterial toxins, namely diphtheria toxin (*Corynebacterium diphtheriae*) and cholix toxin (*Vibrio cholera*; Jørgensen et al., 2008) also promote irreversible ribosylation of human EEF2 on diphthamide 715 (Liu et al., 2004; Ivankovic et al., 2006; Jørgensen et al., 2005). Hence, to determine if those toxins could also induce NLRP1 inflammasome formation in a similar way to that observed with EXOA, we exposed NLRP1 reporter cell lines to those toxins. As for EXOA, we observed that cholix toxin and diphtheria toxin both triggered robust NLRP1 inflammasome complex assembly in those reporter cells (Fig. 2 F). Furthermore, NLRP1 inflammasome formation was abrogated in *DPH1*-deficient A549 in response to cholix toxin and diphtheria toxin, hence confirming the existence of a shared pathway for EEF2 inactivation by those toxins (Fig. 2 F).

Finally, exposure of primary WT and NLRP1-deficient corneal epithelial cells to EXOA highlighted increased protection of *NLRP1*^*−/−*^ cell to cell pyroptosis, suggesting that extracellular *P. aeruginosa–*induced NLRP1 inflammasome response in epithelial cells requires T2SS-secreted EXOA and subsequent EEF2 inactivation (Fig. 2 G).

### EEF2 inactivation drives ribotoxic stress response (RSR)-dependent ZAKα and P38 MAPkinase activation and subsequent NLRP1 inflammasome nucleation

**Figure 3.**
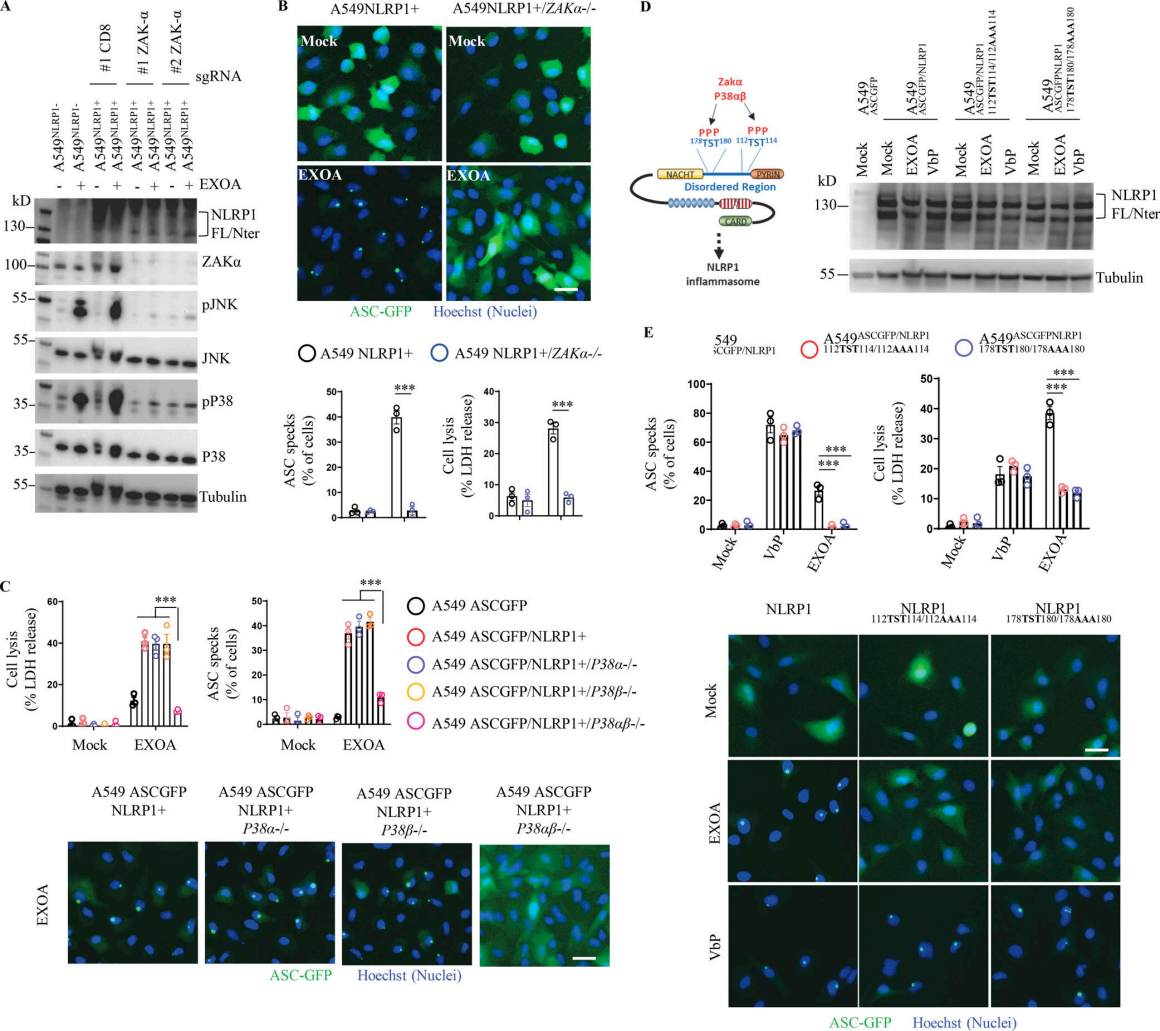
**EEF2 inactivation drives ZAKα and P38 MAPK activation and subsequent NLRP1 inflammasome nucleation. (A)** Immunoblotting of P38, JNK, ZAKα, NLRP1, Tubulin, and phosphorylated P38 and JNK in A549^NLRP1+^ and A549^NLRP1+/ZAKα-^ reporter cell lines exposed or not to EXOA (10 ng/ml) for 3 h. Immunoblots show lysates from one experiment performed at least three times. **(B)** Cell lysis (LDH release), florescence microscopy, and associated quantifications of ASC-GFP specks in A549^NLRP1+/ASC-GFP^ and A549^NLRP1+/ASC-GFP/ZAKα-^ reporter cell lines exposed to EXOA (10 ng/ml) for 10 h. ASC-GFP (green) pictures were taken in the dish after toxin exposure. Images shown are from one experiment and are representative of *n* = 3 independent experiments; scale bars, 10 µm. ASC complex percentage was performed by determining the ratios of cells positives for ASC speckles (green, GFP) on the total nuclei (Hoechst). At least 10 fields from *n* = 3 independent experiments were analyzed. Values are expressed as mean ± SEM. ***P ≤ 0.001, one-way ANOVA. Graphs show one experiment performed in triplicates at least three times. **(C)** Cell lysis (LDH release), fluorescence microscopy, and associated quantifications of ASC-GFP specks in A549^NLRP1+/ASC-GFP^ and A549^NLRP1+/ASC-GFP/P38α/β-^ reporter cell lines exposed to EXOA (10 ng/ml) for 10 h. ASC-GFP (green) pictures were taken in the dish after toxin exposure. Images shown are from one experiment and are representative of *n* = 3 independent experiments; scale bars, 50 µm. ASC complex percentage was performed by determining the ratios of cells positive for ASC speckles (green, GFP) on the total nuclei (Hoechst). At least 10 fields from *n* = 3 independent experiments were analyzed. Values are expressed as mean ± SEM. ***P ≤ 0.001, one-way ANOVA. Graphs show one experiment performed in triplicate at least three times. **(D)** Western blot examination of NLRP1 using an anti-NLRP1 N-terminal antibody (aa 1–323) in A549^ASC-GFP^ reporter cells reconstituted with hNLRP1 or hNLRP1 plasmid constructs mutated for ^112^TST^114^/^112^AAA^114^ or ^178^TST^180^/^178^AAA^180^ after 4 h exposure to EXOA (10 ng/ml) or VbP (15 µM). Images shown are from one experiment and are representative of *n* = 3 independent experiments. **(E)** Cell lysis (LDH release), fluorescence microscopy, and associated quantifications of ASC-GFP specks in A549^ASC-GFP^ reporter cells reconstituted with hNLRP1 or hNLRP1 plasmid constructs mutated for ^112^TST^114^/^112^AAA^114^ or ^178^TST^180^/^178^AAA^180^ after 10 h exposure to EXOA (10 ng/ml) or VbP (15 µM). ASC-GFP (green) pictures were taken in the dish after toxin exposure. Images shown are from one experiment and are representative of *n* = 3 independent experiments; scale bars, 10 µm. ASC complex percentage was performed by determining the ratios of cells positive for ASC speckles (green, GFP) on the total nuclei (Hoechst). At least 10 fields from *n* = 3 independent experiments were analyzed. Values are expressed as mean ± SEM. ***P ≤ 0.001, two-way ANOVA with multiple comparisons. Graphs show one experiment performed in triplicate at least three times. Source data are available for this figure: SourceData F3.

**Figure S3.**
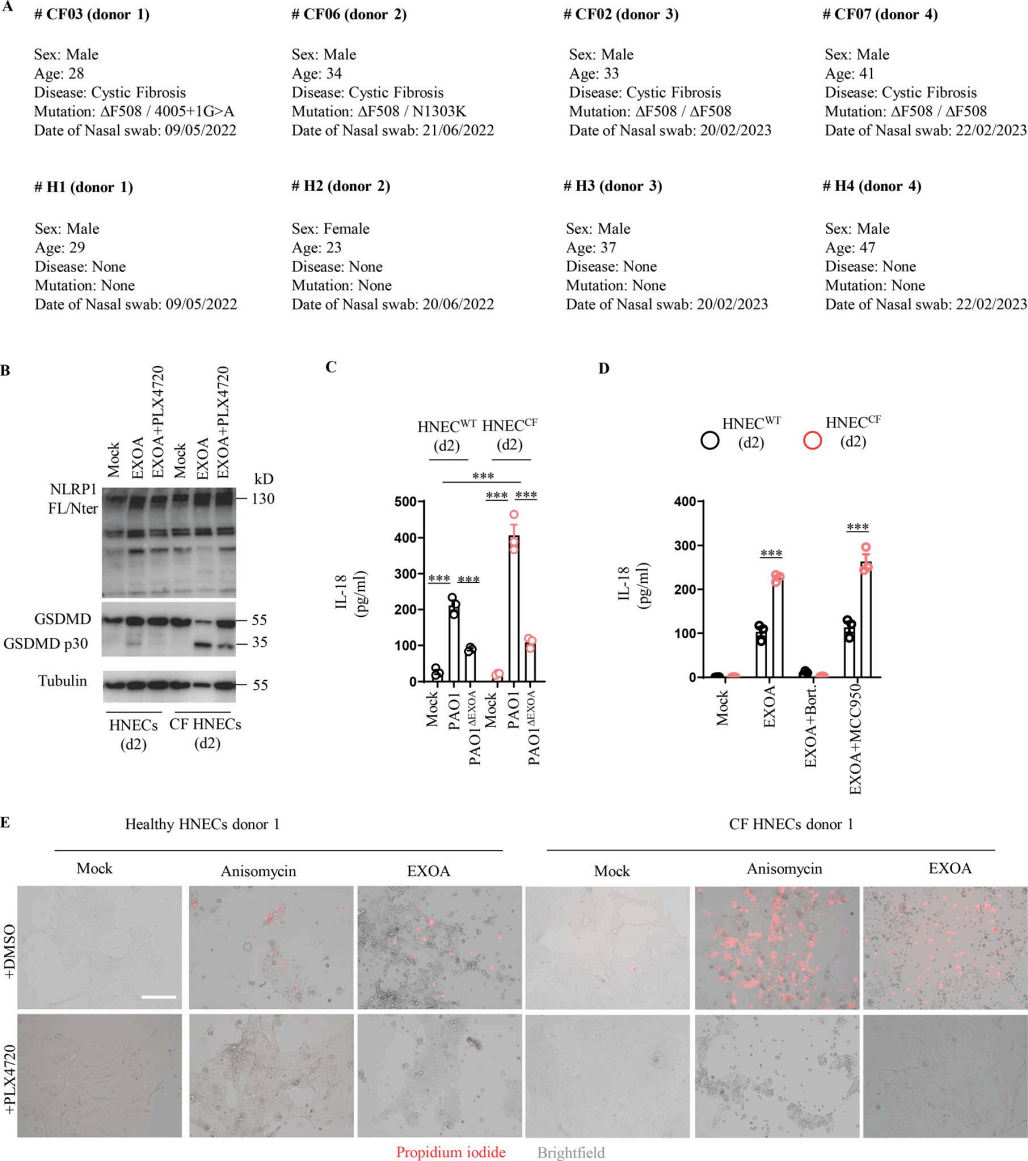
**CF cells show exacerbated sensitivity to undergo cell death upon ribotoxic stress. (A)** Information regarding healthy and CF patient samples used in this study. **(B)** Immunoblotting of NLRP1, Gasdermin-D (GSDMD), and Tubulin in pHNECs^WT^ and pHNECs^CF^ from healthy (WT) and CF patients exposed to EXOA (10 ng/ml) or not for 12 h in presence or absence of PLX420 (ZAKα inhibitor, 10 µM). Immunoblots show combined supernatants and lysates from one experiment performed at least three times. (d2) stands for donor 2 from CF or healthy (WT) patients. Immunoblots show lysates from one experiment performed at least two times. **(C)** IL-18 release in pHNECs^WT^ and pHNECs^CF^ co-cultured with PAO1 or PAO1^ΔEXOA^ (1.10^5^ bacteria) for 24 h. ***P ≤ 0.001, *T* test. Values are expressed as mean ± SEM from one experiment (in triplicate) from one independent donor (d2, CFd2) performed at least three times. **(D)** IL-18 release evaluation in pHNECs^WT^ and pHNECs^CF^ upon EXOA (10 ng/ml), treatment for 18 h in presence/absence of Bortezomib (Bort., proteasome inhibitor, 1 µM) or MCC950 (NLRP3 inflammasome inhibitor, 10 µM). ***P ≤ 0.001, *T* test. Values are expressed as mean ± SEM from one experiment (in triplicate) from one independent donor (d2, CFd2) performed at least two times. **(E)** Fluorescence microscopy of PI (red) incorporation into pHNEC^WT^ (donor 2) and pHNEC^CF^ (donor 1) after exposure to anisomycin or EXOA for 16 h in presence or not of the ZAKα inhibitor PLX4720 (10 µM). Images shown are from one experiment and are representative of *n* = 3 independent experiments; scale bars, 50 µm. Source data are available for this figure: SourceData FS3.

Recent studies unveiled that ribosome inactivation by UV-B, as well as the antibiotic anisomycin, also promotes NLRP1 inflammasome activation in human keratinocytes (Robinson et al., 2022; Jenster et al., 2023; Fenini et al., 2018). Furthermore, this mode of activation engages the MAP3K ZAKα and the effector P38α/β kinases (Robinson et al., 2022; Jenster et al., 2023; Fenini et al., 2018). Thus, we wondered if EXOA-driven EEF2a irreversible inactivation and subsequent NLRP1 inflammasome formation might also engage a similar pathway. We generated ZAKα-deficient reporter cell lines and measured their ability to promote activation of the MAPK stress kinases in response to EXOA exposure (Fig. 3 A and Fig. S3 A). We observed that EXOA, but not its inactive mutant EXOA^H426A^, strongly induced P38 and JNK stress kinase phosphorylation, a process that disappeared in absence of ZAKα (Fig. 3 A and Fig. S2 D). Further analysis of NLRP1 inflammasome formation using fluorescence microscopy highlighted that ZAKα deficiency completely abrogated assembly of the NLRP1 inflammasome and cell death in response to EXOA (Fig. 3 B). To further analyze if ZAKα and its downstream effectors P38 were important for NLRP1 inflammasome formation, we genetically deleted P38α, P38β, or both P38α/β in reporter cells and quantified inflammasome assembly in response to EXOA (Fig. 3 C and Fig. S2 E). We observed that single deletion for P38α or P38β did not strongly modify inflammasome assembly whereas combined deficiency for P38α/β led to a robust defect in NLRP1 inflammasome formation upon EXOA exposure (Fig. 3 C). This suggests that ZAKα- and ZAKα-activated P38 kinases contribute to NLRP1 inflammasome response to EXOA.

ZAKα and P38-driven phosphorylation of serine and threonine in the NLRP1-disordered region has been recently shown to promote NLRP1 inflammasome activation upon UV-B-driven ribosome collision (Robinson et al., 2022). Here, using NLRP1 constructs mutated for either phosphorylation site 1 (^110^TST^112^) or site 2 (^178^TST^180^), we observed that EXOA was unable to trigger assembly of the NLRP1 inflammasome in reporter cells (Fig. 3, D and E). On the contrary, VbP- and SARS-CoV-2-induced NLRP1 inflammasome assembly was fully efficient in cells complemented with either construct or in ZAKα-deficient cells (Fig. 3, D and E; and Fig. S2 D). All in one, these results suggest that ZAKα- and P38-targeted NLRP1 sites 1 and 2 are required for efficient inflammasome assembly in response to EXOA but not during VbP or SARS-CoV-2 exposure.

### CF airway epithelial cells show exacerbated sensitivity to EXOA-driven cell death, which is reversed by ZAKα inhibition

**Figure 4.**
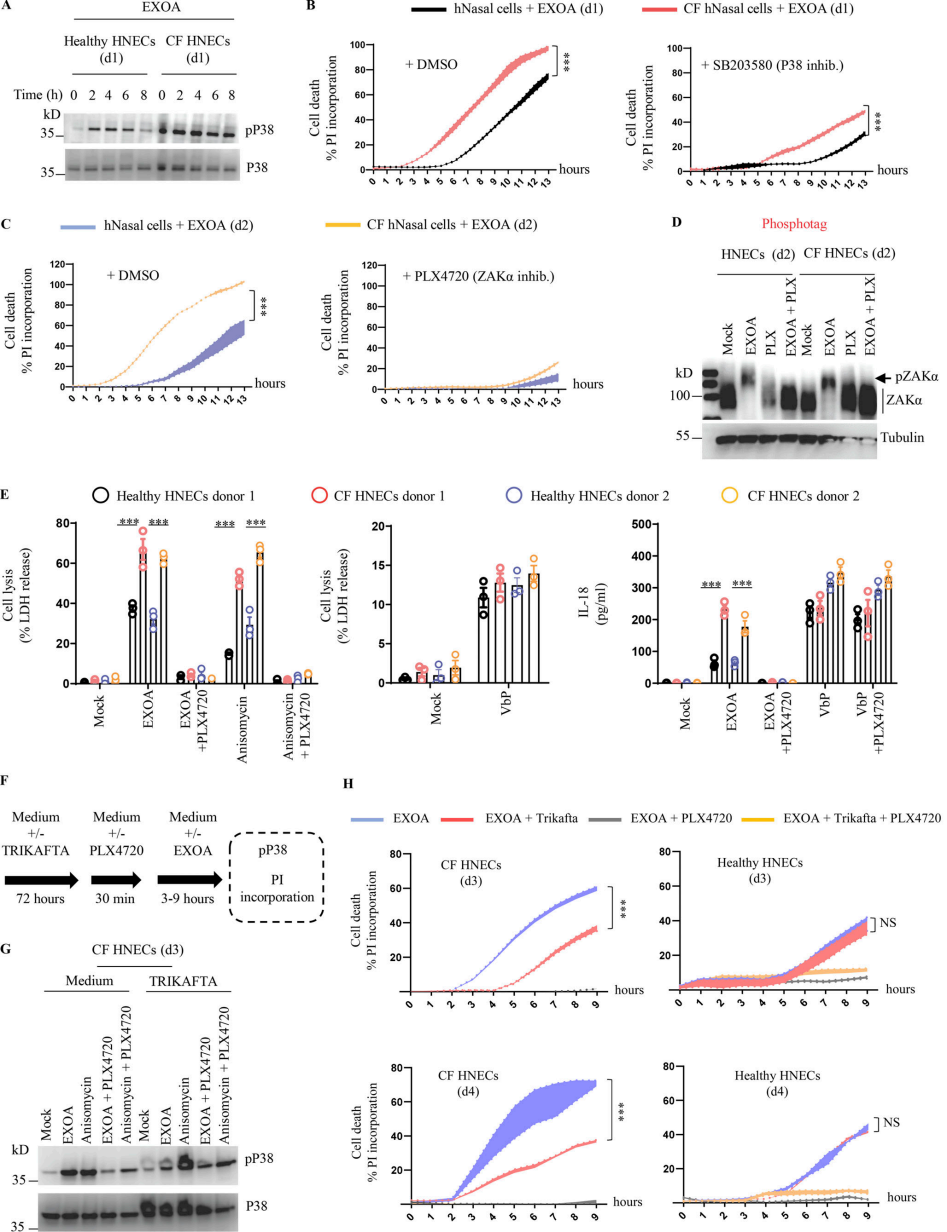
**CF airway epithelial cells show exacerbated sensitivity to EXOA-driven pyroptosis, which is reversed by ZAKα inhibition. (A)** Immunoblotting of P38 and phosphorylated P38 in pHNECs^WT^ and pHNECs^CF^ from healthy (WT) and CF patients exposed to EXOA (10 ng/ml) or not for 8 h. Immunoblots show lysates from one experiment performed at least two times. (d1) stands for donor 1 from CF or healthy (WT) patients. Images shown are from one experiment and are representative of *n* = 2 independent experiments. **(B and C)** Plasma membrane permeabilization determination over time using PI incorporation in pHNECs^WT^ or pHNECs^CF^ exposed to EXOA (10 ng/ml) for indicated times. When specified, SB203580, an inhibitor of P38 activity (10 µM) or PLX420 (bRaf, ZAKα inhibitor, 10 µM) were used. (d1) and (d2) stand for donors 1 or 2, respectively. ***P ≤ 0.001, *T* test. Values are expressed as mean ± SEM from one experiment (in triplicate) from one independent donor (d1/d2, CFd1/CFd2) performed at least three times. **(D)** Phosphotag blotting of phosphorylated ZAKα in pHNECs^WT^ and pHNECs^CF^ from healthy (WT) and CF patients exposed to EXOA (10 ng/ml) or not for 8 h. When specified, PLX420 (bRaf, ZAKα inhibitor, 10 µM) was used. Immunoblots show lysates from one experiment performed at least two times. (d2) stands for donor 2 from CF or healthy (WT) patients. Images shown are from one experiment and are representative of *n* = 3 independent experiments. **(E)** Cell lysis (LDH) and IL-18 release evaluation in pHNECs^WT^ and pHNECs^CF^ upon EXOA (10 ng/ml), anisomycin (1 µg/ml), or VbP (15 µM) treatment for 18 h in presence/absence of PLX420 (ZAK inhibitor, 10 µM). ***P ≤ 0.001, *T* test. Values are expressed as mean ± SEM from one experiment (in triplicate) from one independent donor (d1/d2, CFd1/CFd2) performed at least three times. **(F)** Graphical representation of CFTR correctors Ivacaftor (10 µM), Tezacaftor (10 µM), and Elexacaftor (5 µM) (TRIKAFTA) treatment to CF cells. **(G)** Immunoblotting of P38 and phosphorylated P38 in pHNECs^CF^ treated or not for 72 h with TRIKAFTA and exposed to EXOA (10 ng/ml) or anisomycin (1 µg/ml) for 6 h. Immunoblots show lysates from one experiment performed at least two times. When specified, PLX420 (bRaf, ZAKα inhibitor, 10 µM) was used. Immunoblots show lysates from one experiment performed at least two times. (d3) stands for donor 3 from CF patients. **(H)** Plasma membrane permeabilization determination over time using PI incorporation in pHNECs or pHNECs^CF^ in the presence/absence of TRIKAFTA and subsequently exposed to EXOA (10 ng/ml) for indicated times. When specified, PLX420 (bRaf, ZAKα inhibitor, 10 µM) was used. (d3) and (d4) stand for donors 3 or 4, respectively. ***P ≤ 0.001, *T* test. Values are expressed as mean ± SEM from one experiment (in triplicate) from one independent donor (d3/d4, CFd3/CFd4) performed at least three times. Source data are available for this figure: SourceData F4.

Next, we investigated the role of EXOA-driven pyroptosis in a pathophysiological model of *P. aeruginosa* infection. In addition to causing corneal infections, *P. aeruginosa* is well known to establish life-threatening infection in airways and lungs of CF patients. Due to defective mucus production and clearance (Veit et al., 2016), the airway of CF patients favor chronic *P. aeruginosa* infections. Collaborating with the Hospital of Toulouse, we obtained nasal brushes from four healthy and four CF patients carrying the ∆F508 mutation in the CF transmembrane conductance regulator (CFTR; referred to in the text and figure legends as CF donors 1–4; Fig. S3 A). CF patients have been described to express high basal and inducible P38 kinase activation (Raia et al., 2005; Bérubé et al., 2010), a phenotype we could confirm by exposing those cells to EXOA at various times (Fig. 4 A). Given the importance of P38 kinases in supporting EXOA-dependent NLRP1 inflammasome response, we next measured the ability of healthy and CF nasal cells to undergo EXOA-dependent cell death. Propidium iodide (PI) incorporation measure in healthy and CF nasal cells showed that EXOA-driven cell death was exacerbated in CF nasal cells, a process that was reduced by the use of P38 inhibitor SB203580 (Fig. 4 B). Further experiments also showed that ZAKα inhibition (PLX4720) even further inhibited EXOA-driven cell death, Gasdermin D cleavage, and IL-18 release both in healthy and CF nasal cells (Fig. 4, C–E; and Fig. S3, B and C). However, evaluation of EXOA-driven ZAKα phosphorylation did not show striking differences between healthy and CF cells, suggesting that exacerbated response to EXOA in CF cells occurs downstream of ZAKα activation (Fig. 4 D). As the NLRP3 inflammasome has previously been shown to be strongly activated in CF monocytes, we used the MCC950, an inhibitor of the NLRP3 inflammasome, to evaluate its importance in CF nasal epithelial cells. However, the use of MCC950 did not modify the ability of healthy or CF cells to release IL-18 upon EXOA exposure, suggesting that in primary healthy and CF nasal cells, ZAKα and P38, but not NLRP3, play a major role in response to EXOA (Fig. S3 D).

Importantly, such process was conserved with the ribotoxic antibiotic anisomycin but not in response to VbP where nasal cells from healthy and CF patients responded similarly, hence suggesting that P38 pre- and/or –hyperactivation in CF patients makes them sensitive to ribotoxic stress-driven NLRP1 response (Fig. 4 E and Fig. S3 E).

In this context, we reasoned that if cells from CF patients were more vulnerable to ribotoxic stress-induced cell death, the use of CFTR corrector therapy (TRIKAFTA) could potentially dampen this process. To address this question, we exposed healthy or CF cells to TRIKAFTA for 72 h and evaluated their response to anisomycin- and EXOA-induced P38 phosphorylation and cell death (Fig. 4, F–H). Our results showed that CF cells treated with TRIKAFTA had reduced phosphorylated P38 in response to anisomycin and EXOA (Fig. 4 G). In addition, the exacerbated cell death induced by EXOA in CF cells was strongly decreased in TRIKAFTA-treated CF cells to a level close to EXOA-treated healthy cells, hence suggesting that CF cell sensitivity to ribotoxic stressors can be dampened using TRIKAFTA therapy (Fig. 4 H).

Altogether, our results suggest that the ZAKα/NLRP1 axis contributes to epithelial barrier destabilization upon EXOA-inactivated EEF2 exposure, a process exacerbated in airway epithelial cells from CF patients.

In this study, our findings and those from the Zhong group (companion manuscript; Robinson et al., 2023) highlight that the T2SS-released EXOA and its relatives, cholix and diphtheria toxins, trigger activation of the human NLRP1 inflammasome in skin keratinocytes, corneal, and airway epithelial cells, three important sites of *P. aeruginosa* chronic infections. This process exemplifies a novel biochemical pathway by which epithelial organs detect bacterial virulence factors.

Although major research studies unveiled that upon acute infection, *P. aeruginosa* T3SS allows both activation of the NLRC4 and NLRP3 inflammasomes in rodent as well as in human macrophage and neutrophil models (Sutterwala et al., 2007; Faure et al., 2014; Franchi et al., 2007; Miao et al., 2008; Deng et al., 2015; Balakrishnan et al., 2018; Santoni et al., 2022; Ryu et al., 2017), chronic infections mediated by this pathogen is associated with a downregulation of T3SS in favor of a biofilm phenotype, where EXOA is strongly produced and released. In this regard, our observation that EXOA-driven ribotoxic stress contributes to exacerbated tissue damage and inflammation strongly correlate with earlier studies which showed that EXOA-deficient bacteria triggered lower tissue damage during infections of human and mice (Michalska and Wolf, 2015; Pillar and Hobden, 2002). Even though the RSR-driven NLRP1 inflammasome path is not conserved among rodents and humans, ZAKα-driven RSR constitutes a shared process between both species, which suggests that evolution selected ZAKα as a versatile stress sensor. On the contrary, for yet-to-be-determined reasons, humans link RSR to NLRP1 inflammasome response in epithelia, which suggests specific importance for such a pathway.

A caveat of this work mostly relies on the yet-to-be-determined identification of ubiquitin ligases that promote NLRP1 functional degradation upon ZAKα-driven phosphorylation, which is currently under investigation. The sensitivity of NLRP1 activation to the Nedd8 inhibitor MLN4924 suggests that Nedd8-driven Cullin ligase activation is of major importance in this process (Jenster et al., 2023; Robinson et al., 2022). Among the broad family of Cullin ligases, Cull1 and 2 were found to target phosphorylated proteins for ubiquitination and subsequent degradation (Chen et al., 2021). Should one of those Ubiquitin ligase complexes be involved constitutes an attractive hypothesis to pursue.

In addition to UV-B, Chikungunya virus, the antibiotic anisomycin, and the fungal toxin DON (Fenini et al., 2018; Jenster et al., 2023; Robinson et al., 2022), our work, along the one from Zhong lab, unveils the critical involvement of the NLRP1 inflammasome upon infections mediated by various bacterial pathogens, including *C. diphtheria* and *P. aeruginosa*. In this regard, patients developing CF show exacerbated inflammation and tissue damage upon chronic infection with *P. aeruginosa*. Specific studies highlighted that stress-activated kinases P38 and JNK were overactivated in CF-derived cells from patients (Bérubé et al., 2010; Raia et al., 2005). Although various hypotheses were developed to explain such dysregulation in CF patients, no study, including ours, could unveil the critical molecular and biochemical mechanisms engaged, which warrants further investigations. In addition, an emerging model regarding hNLRP1 activation by phosphorylation suggests that P38 kinases would be essential in this process (Jenster et al., 2023; Robinson et al., 2022). In this regard, it remains enigmatic to us why the strong P38 activity detected in CF cells is not able to directly drive hNLRP1 activity, even in absence of stimulation. One could speculate that during the ribotoxic stress response, ZAKα might drive the activation of additional regulators of P38, that all converge to hNLRP1 inflammasome activation. Another non-exclusive hypothesis would be that below a certain threshold, P38 activity is not sufficient for efficient hNLRP1 activation, a process where host cell phosphatases might play a major role. Independently from this, our findings that in this context, not only EXOA but also other RSR inducers specifically triggered an exacerbated cell death response suggest that targeting ZAKα and/or P38 kinases in *P. aeruginosa*–infected patients might constitute a good host-targeted approach to limit epithelial damages complementary to the current antibiotic and CFTR modulator strategy used (Heijerman et al., 2019).

Finally, it has long been noted that host EEF2 (and EEF1) is targeted by a variety of exotoxins from different unrelated bacterial species. Indeed, EEF2 is highly conserved in all eukaryotic species, all expressing a specific diphtamide amino acid. In this context, yeasts, as well as mammals, are similarly targeted by EXOA and relative toxins, which underlines the outstanding adaptation of *P. aeruginosa* to its environment but also raises the question of the specific species where EXOA holds the most prevalent/potent role for bacterial development/survival. Another key question lies in the recent identification of DPH1 and -2 deficient patients (Urreizti et al., 2020; Nakajima et al., 2018; Hawer et al., 2020). If those deficiencies might actually confer a selective advantage or not to *C. diphtheria*, *V. cholera*, or *P. aeruginosa* infections will constitute an exciting field of investigations, as previously observed for HIV- or malaria-resisting patients (Samson et al., 1996; Allison, 1954), or recently highlighted for *Yersinia pestis*–shaped selection/evolution of the inflammasome-forming sensor PYRIN (Park et al., 2020).

All in one, our results describe the critical role of ZAKα-driven NLRP1 inflammasome response and epithelial disruption in response to the pathogen *P. aeruginosa* and exemplify its deleterious potential in CF pathogenesis.

## Materials and methods

### Ethic statements

Patient data and tissue collection were performed in agreement with the European Network of Research Ethics Committees and French ethic law. The ethical committee, according to the Medical Research Involving Human Subjects Act, reviewed and approved the study. Human tissue was provided by the University Hospital of Toulouse (France) and Centre National de la Recherche Scientifique (agreements CHU 19 244 C and CNRS 205782). All patients involved in this study declared to consent to scientific use of the material; patients could withdraw their consent at any time, leading to the prompt disposal of their tissue and any derived material.

### Reagents used in the study

Reagents used are listed in Table 1.

**Table 1.** List of reagents used in the study

		
**Antibody**	**Catalog reference**	**Provider**
Rabbit anti-ZAK antibody 1:1,000	A301-993A	Bethyl Laboratories
P38 MAPK antibody 1:1,000	9212S	Cell Signaling
Phospho-P38 MAPK (Thr180/Tyr182) (D3F9) XPRabbit mAb 1:1,000	4511S	Cell Signaling
JNK1 Rabbit anti-human polyclonal 1:1,000	44690 G	Invitrogen
Phospho-SAPK/JNK (Thr183/Tyr185) (81E11) Rabbit mAb 1:1,000	4668S	Cell Signaling
Anti-NLRP1 1:200	679802	Biolegend
Anti-NLRP1 (N-terminal) 1:1,000	AF6788-SP;RRID: AB_2916167	R&D Systems
Anti-NLRP1 (C-terminal), 1:1,000	ab36852;RRID: AB_776633	Abcam
Anti-Sheep igG HRP (1/4,000)	HAF016;RRID: AB_562591	R&D
Goat-anti-rabbit IgG (H+L), HRP conjugate (1/5,000)	R-05072-500;RRID: AB_10719218	Advansta
GSDMDC1 antibody 1:1,000	NBP2-33422	Biotechne
Anti-puromycin antibody, clone 12D10	MABE343	Sigma-Aldrich
EEF2 antibody (1:1,000)	2332S	Cell Signaling
DPH1 antibody (1:1,000)	#H00001801-M02	Thermo Fisher Scientific
Anti-α Tubulin antibody 1:1,000	ab4074	Abcam
Streptavidin—HRP	434323	Thermo Fisher Scientific
Anti-NLRP3/NALP3,mAb (Cryo-2)	AG-20B-0014-C100	AdipoGen
**Reagents**	**Catalog reference**	**Provider**
Transwell	140620	Thermo Fisher Scientific
NAD+, biotin-labeled	80610	BPSBioscience
Pseudomonas exotoxin A 0.5 mg	P0184-.5 MG	Sigma
Inactive exotoxin A	N.A.	Creative Diagnostics
Diphtheria toxin	D0564-1MG	Sigma-Aldrich
Cholix toxin	This study	This study
Anisomycin	SE-S7409-10 MG	Selleck
Talabostat (Val-boro-pro, VbP)	tlrl-VbP-10	Invivogen
PhoSTOP	4906845001	Sigma-Aldrich/Roche
cOmplete, Mini, EDTA-free protease inhibitor cocktail	4693159001	Sigma-Aldrich/Roche
Lipofectamine LTX	15338030	Invitrogen
Puromycin dihydrochloride	A1113803	Gibco
Molecular probes SYTOX Green nucleic acid stain	S7020	Thermo Fisher Scientific
Propidium iodide	P1304MP	Thermo Fisher Scientific
Nate	lyec-nate	Invivogen
Phos-tag Acrylamide	AAL-107	Wako Chemicals
Manganese chloride(II)	63535	Sigma-Aldrich
Prestained protein size marker III	230-02461	Wako Chemicals
Ivacaftor (VX-770) 5 mg/10 µM	S1144	Selleck
Tezacaftor (VX-661) 5 mg/10 µM	S7059	Selleck
Elexacaftor (VX-445) 5 mg/5 µM	S8851	Selleck
**Critical commercial assays**	**Catalog reference**	**Provider**
Human total IL-18 duo set	DY318-05	R&D
Cyquant LDH	C20301	Thermo Fisher Scientific
Human IL-1β	88-7261-77	Thermo Fisher Scientific
Q5 site-directed mutagenesis kit protocol	E0554	NEB
**Chemicals**	**Catalog reference**	**Provider**
ZAK inhibitor (PLX-4720) 10 µM	HY-51424	Med Chem Express
P38 inhibitor SB 203580 10 µM	HY-10256	Med Chem Express
Caspase-1 inhibitor—Ac-YVAD-cmk 20 µM	inh-yvad	Invivogen
Z-VAD-FMK 20 µM	vad-tlrl:	Invivogen
Caspase-3/7 inhibitor ZDEVD-FMK 20 µM	S7312	Selleck
Caspase-8 inhibitor - Z-IETD-FMK	inh-ietd	Invivogen
Bortezomib 1 µM	S1013	Selleck
CHX	C4859-1Ml	Sigma-Aldrich
MCC950, 10 mg	Inh-mcc	Invivogen
**hPNECs**	**Catalog reference**	**Provider**
Tryple express enzyme (1X), no phenol red	10718463	Thermo Fisher Scientific
Nutragen type I collagen solution, 6 mg/ml (Bovine)	5010-50 Ml	Advanced Biomatrix
Sputolysin	560000-1SET	Sigma-Aldrich
PneumaCult-Ex plus medium	05040	Stem Cell
Costar 6.5 mm transwell, 0.4 µm pore polyester membrane inserts	38024	Stem Cell
**hPCECs**	**Catalog reference**	**Provider**
Human corneal epithelial cell growth medium	221-500	Tebu-bio
Human corneal epithelial cells	630-05a	Tebu-bio
**Cell lines**	**Catalog reference**	**Provider**
A549 ASC GFP NLRP1	a549-ascg-nlrp1	Invivogen
A549 ASC GFP	a549-ascg	Invivogen
A549 ASC GFP NLRP1/ACE2	a549-ascov2-nlrp1	Invivogen
A549 ASC GFP ACE2	a549-ascov2	Invivogen
THP1	thp-null	Invivogen
**Recombinant DNA**	**Catalog reference**	**Provider**
hNLRP1 gene	puno1-hnalp1a	Invivogen

### Cell culture

A549 cells were maintained in Dulbecco’s modified Eagle’s medium (DMEM; Gibco) supplemented with 10% heat-inactivated fetal bovine serum, 1% penicillin-streptomycin, and 1% *L*-glutamine at 37°C 5% CO_2_. pHCECs were maintained in Human Corneal Epithelial Cell Growth Medium (Tebu-bio) at 37°C 5% CO_2_.

Regarding pHNECs, patients’ pHNECs were collected on superior turbinates using smear brushes at the Hospital of Toulouse, France. After brushing back cells in collection medium, centrifugation was performed for 5 min 400 *g* at 4°C. Pellet was resuspended in 4 ml TrypLE express (GIBCO) + 20 µl Sputolysin (200X) and incubated at 37°C for 5 min to disrupt mucus. TrypLE was diluted with 4 ml of Advanced DMEM F12-.

Pellet was recovered after centrifugation and culture was continued in the expansion medium Pneumacult. After a week of proliferation, basal cells were counted and seeded onto collagen-coated (0.03 mg/ml) and maintained in Pneumacult Ex Plus Medium (StemCell) at 37°C 5% CO_2_.

### Cell stimulations

Otherwise specified, cells were plated 1 d before stimulation in 12-well plates at 200,000 cells per well in 1 ml of DMEM, 10% FCS, and 1% penicillin/streptomycin.

Medium was changed to OPTIMEM and cells were preincubated or not with the indicated inhibitors for 1 h.

The pHNECs were seeded the day before the stimulation in 12-well plates at 200,000 cells per well in 1 ml of Pneumacult medium. Cell’s medium was changed to OPTIMEM and cells were treated or not with the indicated inhibitors for 1 h.

The pHCECs were seeded the day before the stimulation in 12-well plates at 10,000 cells per well in 1 ml of corneal epithelial cell growth medium. Medium of the cells was changed to OPTIMEM and cells were treated or not with the indicated inhibitors for 1 h.

All cells were treated with the indicated concentration of EXOA (10 ng/ml to 1 µg/ml), anisomycin (1 µM), or with VbP (15 µM) for indicated times.

SARS-CoV-2 (BetaCoV/France/IDF0372/2020 isolate) experiments were performed in a BSL-3 environment as described in Planès et al. (2022).

Briefly, 250,000 A549^ASC-GFP/NLRP1/ACE2^ genetically invalidated or not for ZAKα, were infected for 24 h with BetaCoV/France/IDF0372/2020 strain at indicated multiplicity of infection (MOI) in DMEM supplemented with 10 mM Hepes, 1% penicillin-streptomycin, and 1% *L*-glutamine for 1 h at 37°C.

For rescue experiments, a combination of three CFTR correctors Ivacaftor (10 µM), Tezacaftor (10 µM), and Elexacaftor (5 µM; TRIKAFTA) was used for 72 h on CF nasal cells from patients before stimulation with EXOA or anisomycin as described above.

### Transwell infections

pHNECs, pHCECs, and A549 cells, expressing or not NLRP1, along with the ASC-GFP reporter were plated in 24-well plates at 2.5 × 10^5^ cells per well 1 d before experiment. The following day, cell culture media was replaced by 600 µl of OPTIMEM per well. Cells were placed in co-culture with *P. aeruginosa* at MOI of 10 in 100 µl of OPTIMEM separated by a semipermeable transwell insert (0.3 µm). After 18 h of co-culture, transwell inserts containing bacteria were removed and ASC-speck formation in A549 cell was analyzed. Images were acquired using EVOS M700 microscope.

When specified, CFU determination was addressed by flushing three times transwell membranes with PBS, and serial dilutions of bacteria were performed on Luria Broth (LB)–agar plates for CFU determination.

### ASC specks imaging and quantifications

ASC-speck formation in A549 cells was imaged by using EVOS 7000 microscope with a 20× magnificence. Quantification and analysis of ASC aggregates (or specks) were performed by determining the ratios of ASC aggregates (i.e., ASC speck) formed in each cell over the total nuclei numbers (staining with Hoechst 33342) by using Image J software. Quantifications were performed in a blinded way.

### Bacterial growth and mutants

*P. aeruginosa* strains (PAO1, clinical isolates) and their isogenic mutants were grown in LB medium overnight at 37°C with constant agitation. The following day, bacteria were subcultured by diluting overnight culture 1:25 and grown until reaching an optical density (OD) OD600 of 0.6–0.8.

PAO1 and its transposon mutants were obtained from a two-allele transposon library (Jacobs et al., 2003; Table 2).

**Table 2.** List of bacterial strains used in this study from the transposon library (Jacobs et al., 2003)

Name	Exact	Location	ORF	Gene abbrev.
PW3079	+	phoAwp05q1C05	PA1148	toxA (EXOA)
PW2538	+	phoAwp03q1H07	PA0844	plcH (PLCH)
PW6586	+	phoAwp08q2E01	PA3319	plcN (PLCN)
PW6221	+	lacZwp03q2D06	PA3105	xcpQ (T2SS)
PW7302	+	phoAbp02q4F10	PA3724	LasB (LASB)
PW4017	+	lacZbp02q3F02	PA1706	pcrV (T3SS)
PW10311	+	lacZbp02q1B09	PA5503	ABC transporter (T1SS)

Specific deletion of xcpQ (T2SS) and toxA (EXOA) genes was achieved as described previously in Santoni et al. (2022). Briefly, the pEXG2 suicide vector containing 700 bp sequences of the flanking regions of the selected gene was directly inserted into competent SM10λpir (Mix&Go competent cells; Zymo Research Corporation) and subsequently selected on LB-agar supplemented with 50 μg/ml kanamycin/15 μg/ml gentamicin. After sequencing, the resulting clones were mated with PAO1 strains, 4H/37°C. Mated bacteria were plated on 15 μg/ml gentamicin and 20 μg/ml Irgasan LB-agar plates to selectively remove *Escherichia coli* SM10 strains. The next day, 5–10 clones were grown for 4 h in LB and streaked on 5% sucrose LB plates overnight at 30°C. PAO1 clones were then checked by PCR for mutations (Table 3).

**Table 3.** Primer sequences used for the generation of various genetic deletions in PAO1 strains

Name	Sequence	Targeted gene
607_JB_PA_m_toxA_R1 Fw	5′-CCC​AGT​CTC​GAG​GTC​GAC​GGT​ATC​GAT​AAG​CTT​GAT​ATC​GAA​TTC​GGC​CGA​CGG​CGG​C-3′	toxA
608_JB_PA_m_toxA_R1 Rv	5′-TCG​CGA​TGC​ACC​TGA​CAC​CCG​AGG​ACC​TGA​AGT​AAC​TGC​CGC-3′	toxA
609_JB_PA_m_toxA_R2 Fw	5′-GGC​AGT​TAC​TTC​AGG​TCC​TCG​GGT​GTC​AGG​TGC​ATC​GC-3′	toxA
610_JB_PA_m_toxA_R2 Rv	5′-CTG​GAG​CTC​CAC​CGC​GGT​GGC​GGC​CGC​TCT​AGA​ACT​AGT​GGA​TCC​AGC​CAT​TGT​TCG​ACG​AAT​AAA​GCC​ACC-3′	toxA
611_JB_PA_m_toxA_Check R1	5′-GAA​GTA​CTT​CAA​CGG​GTT​GAT​CCC​C-3′	toxA
612_JB_PA_m_toxA_Check R2	5′-CGT​TTC​CGC​AAC​GCT​TGA​AGT-3′	toxA
EM019-xcpQ R1 Fw	5′-TTC​CAC​ACA​TTA​TAC​GAG​CCG​GAA​GCA​TAA​ATG​TAA​AGC​AAG​CTT​ACG​ATA​AAG​ACC​AGG​AGT​GAT​GTA​TTG​CC-3′	XcpQ
EM020-xcpQ R1 Rv	5′-CGC​CGT​TAT​TCC​GTC​ATC​AGC​AAA​GGC​TGG​GAC​ATC​GG-3′	XcpQ
EM021-xcpQ R3 Fw	5′-ACC​CGA​TGT​CCC​AGC​CTT​TGC​TGA​TGA​CGG​AAT​AAC​GGC​GCC-3′	XcpQ
EM022-xcpQ R3 Rv	5′-GGA​AAT​TAA​TTA​AGG​TAC​CGA​ATT​CGA​GCT​CGA​GCC​CGG​GGA​TCC​ACG​CCT​GGT​TCG​TGG​C-3′	XcpQ
EM023-xcpQ R1 check	5′-CCC​GGC​CAG​TCA​CAC​CTA​TTG​AT-3′	XcpQ
EM024-xcpQ R3 check	5′-AGT​GCT​TGC​CGA​CAA​CGA​CC-3′	XcpQ

### Cell death assays

Cell lysis was measured by quantification of the LDH release into the cell supernatant using the LDH CyQUANT kit (Thermo Fisher Scientific). Briefly, 50 µl cell supernatant was incubated with 50 µl LDH substrate and incubated for 30 min at room temperature protected from light. The enzymatic reaction was stopped by adding 50 µl of stop solution. Maximal cell death was determined with whole cell lysates from unstimulated cells incubated with 1% Triton X-100.

### Plasma membrane permeabilization assays were performed using PI incorporation

Cells were plated at a density of 1 × 10^5^ per well in Black/Clear 96-well Plates in OPTIMEM culture medium supplemented with PI dye (1 µg/ml) and infected/treated as mentioned in figure legends. Red fluorescence is measured in real-time using a Clariostar plate reader equipped with a 37°C cell incubator or using an EVOS Floid microscope (Invitrogen). Maximal cell death was determined with whole cell lysates from unstimulated cells incubated with 1% Triton X-100.

### Cytokine quantification

Cytokine secretion was quantified by ELISA kits according to the manufacturer’s instructions, IL-1B (88-7261-77; Thermo Fisher Scientific Scientific) and IL-18 (DY318-05; Biotechne).

### Sample preparation for immunoblot

At the end of the experiment, cell supernatant was collected and soluble proteins were precipitated using trichloroacetic acid as described previously (Santoni et al., 2022). Precipitated pellet was then resuspended in 50 µl of radioimmunoprecipitation assay (RIPA) buffer (150 mM NaCl, 50 mM Tris-HCl, 1% Triton X-100, 0.5% Na-deoxycholate) supplemented with protease inhibitor cocktail (Roche). Adherent cells were lysed in 50 µl of RIPA buffer supplemented with protease inhibitor cocktail (Roche). Cell lysate and cell supernatant were homogenized by pipetting up and down 10 times and supplemented with Laemli buffer (1X final) before boiling the sample for 10 min at 95°C.

### Immunoblot

Cell lysates were separated by denaturing SDS-PAGE and transferred to the polyvinylidene fluoride membrane. After transfer, the membrane was saturated for 1 h at room temperature in TBS-T (Tris 10 mM, pH 8, NaCl 150 mM, Tween 20 0.05%) containing 5% BSA. Then, the membrane was incubated overnight at 4°C with the different primary antibodies under agitation. After three washes with TBS-T, the membrane was further incubated with the secondary antibodies coupled with the peroxidase enzyme HRP for 1 h at room temperature and under agitation. Then membranes were washed three times with TBS-T. ECL revelation kit (Advansta) was used as a substrate to reveal HRP activity and membranes are imaged using ChemiDoc Imaging System (BioRad). The primary antibodies and secondary antibodies used are listed in Table 1.

### Phosphoblots

At the end of the experiment, cell supernatant was discarded and adherent cells were lysed in 50 µl of RIPA buffer (150 mM NaCl, 50 mM Tris-HCl, 1% Triton X-100, 0.5% Na-deoxycholate) supplemented with protease inhibitor cocktail (Roche) and phosphatase inhibitors cocktails (Roche). Collected cell lysate was homogenized by pipetting up and down 10 times and supplemented with Laemli buffer before boiling for 10 min at 95°C. Cell lysates were then separated by SDS-PAGE and handled as described in the immunoblot section.

### Phosphotags

At the end of the experiment, cell supernatant was discarded and adherent cells were lysed in 50 µl of RIPA buffer supplemented with Laemli buffer and protease and phosphatase inhibitor cocktails. Samples were then boiled for 10 min at 95°C. Cell lysates were separated by PhosTag SDS-PAGE and the size of proteins was determined by the following size marker: wide-view prestained protein size marker III (Wako Chemicals). PhosTag SDS-PAGE was carried out using homemade 10% SDS-PAGE gel, with addition of Phos-tag Acrylamide (AAL-107; Wako Chemicals) to a final concentration of 30 μM and manganese chloride(II) (63535; Sigma-Aldrich) to 60 µm. Once the run was completed, the polyacrylamide gel was washed in transfer buffer with 10 mM EDTA twice, subsequently washed without EDTA twice, and transferred to polyvinylidene fluoride membranes, thanks to a Trans-Blot Turbo (Bio-rad). Membranes were blocked at 4°C with 5% milk and incubated overnight at 4°C with primary and corresponding secondary antibodies at room temperature.

### Puromycin incorporation

The day before the stimulation, A549 ASC-GFP expressing or not NLRP1 were seeded in 12-well plates at 200,000 cells in 1 ml of DMEM, 10% FCS, 1% penicillin/streptomycin. Cells were treated with EXOA (10 ng/ml) for indicated times. 30 min before the end of the treatment, puromycin antibiotic was added in cell medium at 1 µg/ml final. Following puromycin incubation, supernatant was discarded and adherent cells were prepared for immunoblot as described in the Immunoblot section. Puromycin incorporation was revealed using the following antibody anti-puromycin antibody (clone 12D10 MABE343).

### Polysomes profiling

#### Sucrose gradient preparation

Five sucrose solutions containing 10%, 20%, 30%, 40%, and 50% sucrose (wt/vol) were prepared in TMK buffer (20 mM Tris-HCl, pH 7.4, 10 mM MgCl2, 50 mM KCl). Layers of 2.1 ml of each solution were successively poured into 12.5 ml polyallomer tubes (Cat. # 331372; Beckman-Coulter) starting from the most concentrated solution (50%) at the bottom of the tubes to the least concentrated solution (10%) at the top. Each layer was frozen in liquid nitrogen before pouring the following one. Frozen gradients were stored at −80°C and slowly thawed overnight at 4°C before use. For extract preparation, A549 cells were grown to 70% confluency and then treated or not with EXOA (10 ng/ml) for indicated times. Following this treatment, cells were incubated with cycloheximide (CHX, 100 µg/ml) for 15 min at 37°C. Following treatment, cells were rinsed twice with PBS and treated with 1 ml of trypsin (0.25%) for 5 min at 37°C. Trypsin was diluted with DMEM medium containing 100 µg/ml of CHX. Cells were mixed up and down and counted to adjust the final resuspension volume in each condition. Cells were centrifuged at 1,200 rpm (300 *g*) for 5 min at 4°C. The cell pellet was washed with ice-cold PBS containing 100 µg/ml of CHX. Cells were centrifuged at 1,200 rpm (300 *g*) for 5 min at 4°C. Supernatants were aspirated and gently lysed in lysis buffer (20 mM Tris-Cl [pH 8], 150 mM KCl, 15 mM MgCl2, 1% Triton X-100, 1 mM dithiothreitol, 100 µg/ml CHX, EDTA-free and protease inhibitor cocktail). Samples were incubated on ice for 20 min and centrifuged at 1,000 *g* for 5 min at 4°C min and supernatant corresponding to the cytosolic fraction of the cells was collected into a 1.5-ml tube. Samples were further centrifugated at 10,000 *g* for 5 min at 4°C to clarify the cytoplasmic extract. Extracts were quantified by measuring absorbance at 260 nm using Nanodrop.

### Extracts loading, gradient centrifugation, and collection

Normalized amounts of extracts were loaded on 10–50% sucrose gradients and then centrifuged at 260,800 × *g* for 2.5 h at 4°C in an Optima L-100XP ultracentrifuge (Beckman–Coulter) using the SW41Ti rotor with brake. Following centrifugation, fractions were collected using a Foxy R1 gradient collector (Teledyne Isco) driven by PeakTrak software (Version 1.10; Isco Inc.). The A_254_ was measured during collection with a UA-6 UV/VIS DETECTOR (Teledyne Isco). The final polysome profiles were generated in Excel from .txt files extracted from PeakTrak software.

### EEF2 ribosylation assays

Cell lysates from A549 were prepared by suspending one pellet of 1 × 10^6^ A549 cells in 100 µl of RIPA buffer. For in vitro ADP-ribosylation assays, reactions were performed in Eppendorf tube by mixing 50 µl of whole cell lysate with 100 µl of ADP-ribosylation buffer (20 mM Tris-HCl [pH 7.4], 150 mM NaCl, and 1 mM dithiothreitol) supplemented with NAD+ Biotin-Labeled (BPSBioscience) at 50 µM final, in presence or absence of recombinant EXOA protein (100 ng). Reaction was left for 1 h at 25°C. 20 µl of the reaction mixture was analyzed by SDS-PAGE followed by Western blotting as described in the Immunoblot section. Membranes were first subjected to EEF2 detection (using anti-EEF2 followed by HRP-conjugated secondary antibody), then membranes were stripped, and ADP ribosylation was detected by monitoring the incorporation of the NAD+ Biotin-Labeled probe using streptavidin-HRP-conjugate.

### Generation of mutations in NLRP1 gene

To generate NLRP1 gene mutated for each phosphorylation site (site 1 [^110^TST^112^] or site 2 [^178^TST^180^]), threonines and serines were substituted with alanine in the human NLRP1 gene (isoform 1) by site-directed mutagenesis using Q5 site-directed mutagenesis Kit Protocol (E0554) according to the manufacturer’s instructions. Primers used are listed in Table 4.

**Table 4.** Primer sequences used for the generation of mutations in the h*NLRP1* gene

Name	Sequence	T°C
Primer FWD site 1 (3406-3414)	5′-CGC​CGC​AGT​GCT​AAT​GCC​CTG​G-3′	68°C
Primer RV site 1	5′-GCG​GCG​GGT​TGG​CTG​GGA​GAC​CC-3′	68°C
Primer FWD site 2 (3604-3612)	5′-CGC​AGC​AGT​GCT​GGG​GAG​CTG​G-3′	72°C
Primer RV site 2	5′-GCT​GCG​GGG​GCG​TTG​GGT​GAC​TC-3′	72°C

### Cell transfection

The day prior to transfection, A549 cells were plated in a 6-well plate at 2 × 10^5^ cells per well in 1 ml of DMEM complete medium. The following day, cells were incubated with Nate 1X (Invivogen) for 30 min. 1 µg of NLRP1 plasmids (pLvB72 hNLRP1, pLvB72 hNLRP1 [3604-3612], and pLvB72 hNLRP1 [3406-3414]) were transfected using lipofectamine LTX and PLUS reagent according to the manufacturer’s instructions (Invitrogen). Transfected cells were incubated for 48 h before further treatments.

### Cholix toxin production

Recombinant cholix toxin was produced using an adapted methodology from Ogura et al. (2011). Briefly, BL21 (DE3) *E. coli* expressing cholix toxin with a N-terminus hexahistidine-MBP-tag was harvested and bacteria were lysed by sonication on ice. Recombinant cholix toxin was first purified by nickel metal affinity chromatography (Takara) and subsequent TEV protease-mediated 6His-MBP tag removal at 4°C overnight (ON). Then, a second nickel metal affinity chromatography allowed harvesting cholix toxin in the flow-through fraction.

### Genetic invalidations

Genetic invalidation of NLRP1 in pHNECs and pHCECs was achieved by using RNP technic and nucleofection as described previously (Planès et al., 2022). RNP mixes containing Cas9 protein (90 pmoles, 1081059; IDT), gRNA (450 pmoles), and electroporation enhancer (1 μl/Mix, 1075916; IDT) were electroporated using the Neon transfection system (Life Technologies) in T Buffer (Life Technologies). Settings were the following: 1900 V Voltage, 10 Width, 1 Pulse, 20 ms.

Efficient NLRP1 targeting single-guide (sg)RNA sequence was provided by F.L. Zhong (5′-GAT​AGC​CCG​AGT​GCA​TCG​G-3′; Robinson et al., 2022).

Regarding genetic invalidation of P38 isoforms and ZAKα, A549 cells were transduced with LentiCRISPR-V2 vectors containing sgRNA guides against DPH1, P38 isoforms, and ZAKα. 48 h after transduction, cells were selected for 2 wk and puromycin double–resistant cells were used in functional assays. A second round of lentiviral infection with lenti-Blasticidin-sgP38β in single KO cells for P38α achieved generation of double KO cells for P38α and P38β. Cells were subsequently selected in blasticidin antibiotic before checking genetic invalidation by immunoblotting.

Guides were used to generate genetic invalidation (Table 5).

**Table 5.** Single RNA guides used to generate genetic invalidation

Name	Sequence	Direction
P38 α (MAPK14)	5′-AGG​AGA​GGC​CCA​CGT​TCT​AC-3′	FWD
P38 β (MAPK11)	5′-GCC​CTC​GCG​CCG​GCT​TCT​AC-3′	FWD
ZAKα (MAP3K20)	5′-TGT​ATG​GTT​ATG​GAA​CCG​AG-3′	FWD
Dph1	5′-GTT​CAC​GGA​GGC​CGA​AGT​GA-3′	FWD
CD8	5′-TCG​TGG​CTC​TTC​CAG​CCG​CG-3′	FWD

### Analysis

Prism 8.0a (GraphPad Software, Inc.) was used to perform statistical analysis. All relevant information is included directly in figure legends. Otherwise written, data are reported as mean with SEM. Regarding the comparison between two groups, *T* test with Bonferroni correction was chosen and multiple group comparisons were analyzed by using two-way ANOVA with multiple comparisons test. P values are shown in figures and are linked to the following meaning: NS, non-significant, and significance is specified as *P ≤ 0.05; **P ≤ 0.01, ***P ≤ 0.001.

### Online supplemental material

Fig. S1 shows the ability of clinical isolates of *P. aeruginosa* to activate the hNLRP1 inflammasome. Fig. S2 shows the ability of EXOA at promoting ribotoxic stress in epithelial cells as well as the different pathways leading to hNLRP1 inflammasome response upon EXOA exposure or SARS-CoV-2 infection. Fig. S3 supports Fig. 4 findings on the sensitivity of CF cells to ribotoxic stress induction by EXOA.

## Supplementary Material

SourceData F1 is the source file for Fig. 1.

SourceData F2 is the source file for Fig. 2.

SourceData F3 is the source file for Fig. 3.

SourceData F4 is the source file for Fig. 4.

SourceData FS1 is the source file for Fig. S1.

SourceData FS2 is the source file for Fig. S2.

SourceData FS3 is the source file for Fig. S3.

## Data Availability

The data underlying Figs. 1, 2, 3, and 4 and Figs. S1, S2, and S3 are available in the published article and its online supplemental material.
