# Supplementary figures and images for: EEF2-inactivating toxins engage the NLRP1 inflammasome and promote epithelial barrier disruption
Source: J Exp Med. 2023 Aug 29;220(10):e20230104. doi: 10.1084/jem.20230104 (PMC10465324; doi:10.1084/jem.20230104)

SourceDataF1

B

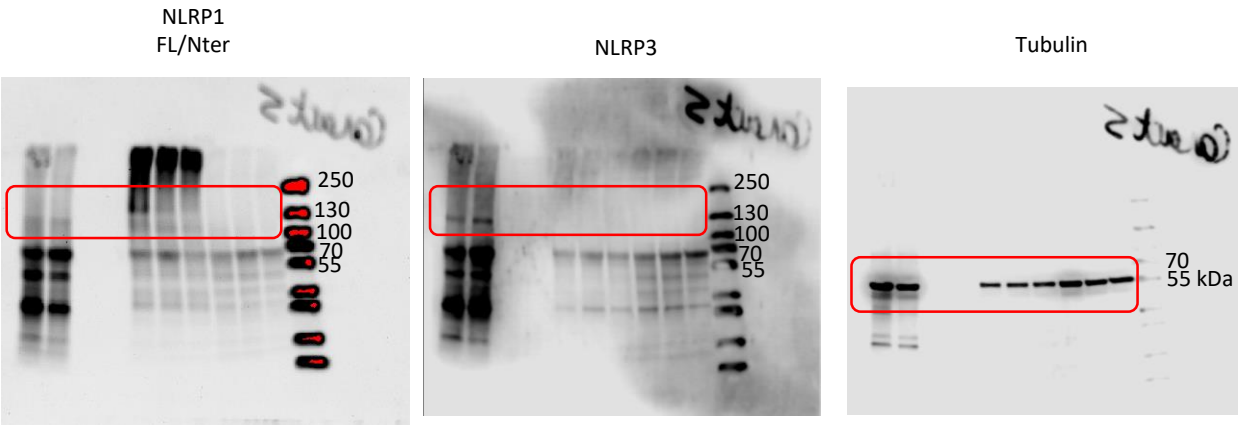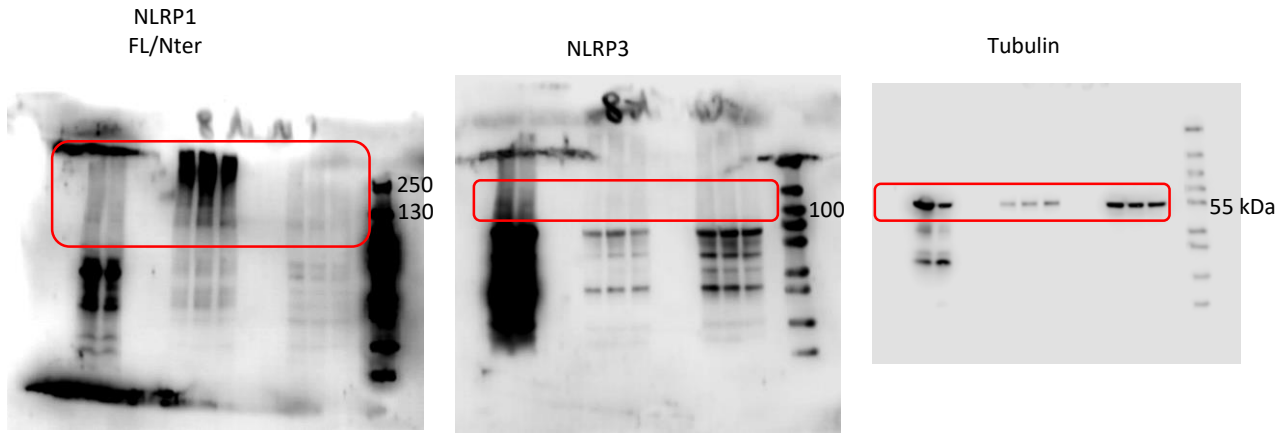

D

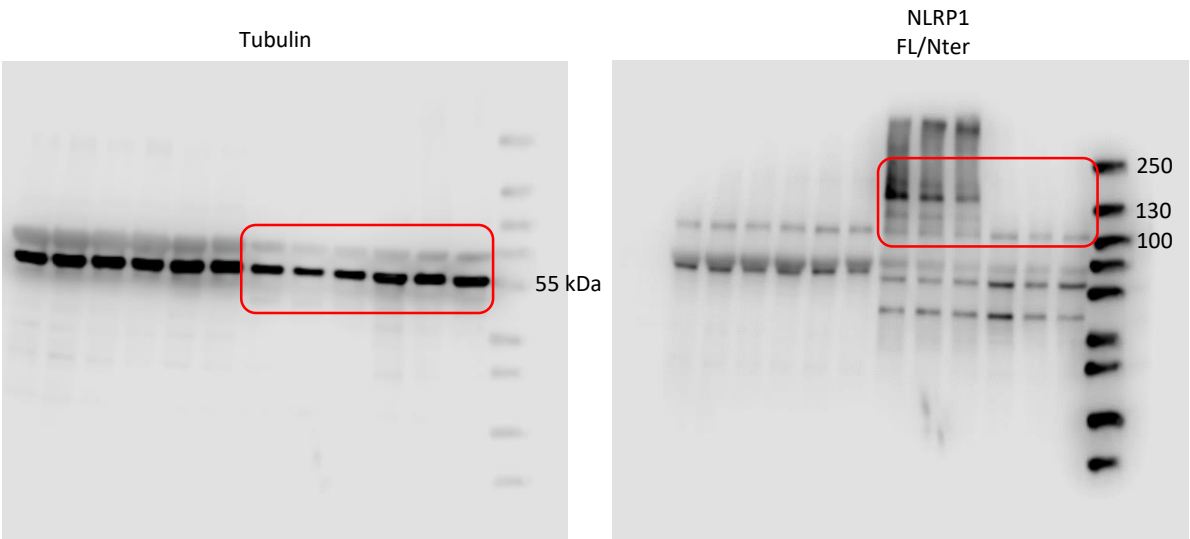

Supplement: Supplementary file 1 — SourceData F1 is the source file for Fig. 1. [file JEM_20230104_SourceDataF1.pdf]

E

DPH1

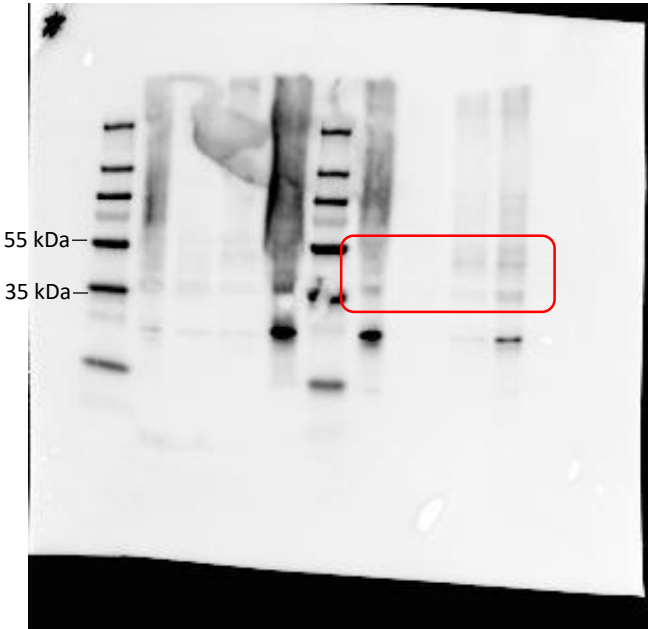

Actin

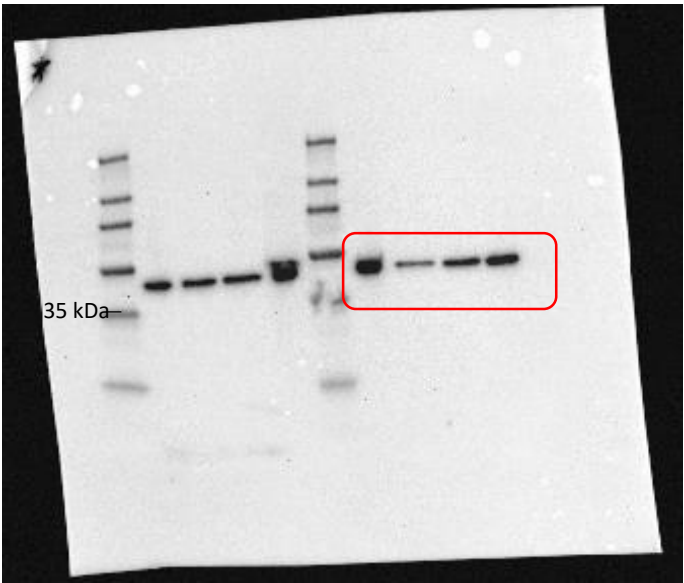

Supplement: SourceData F2 — is the source file for Fig. 2. [file JEM_20230104_SourceDataF2.pdf]

A

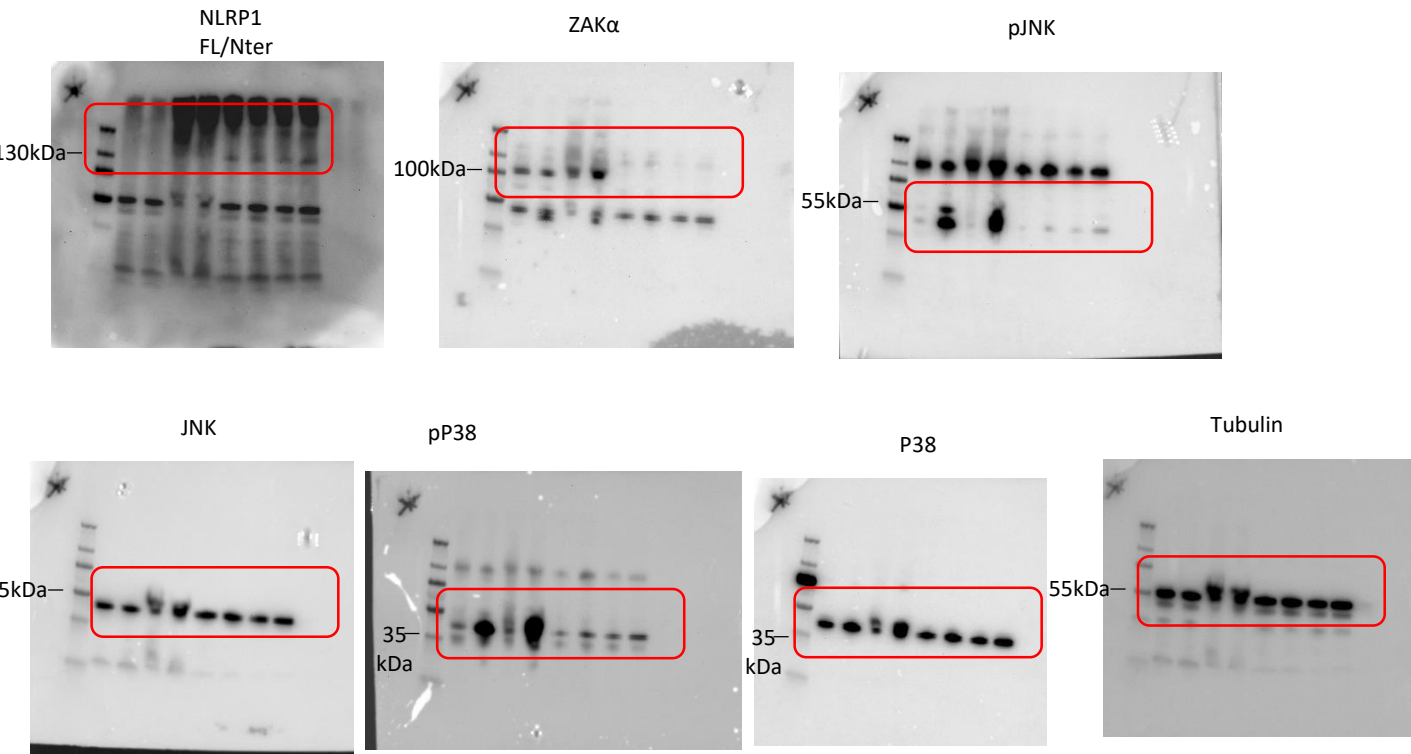

D

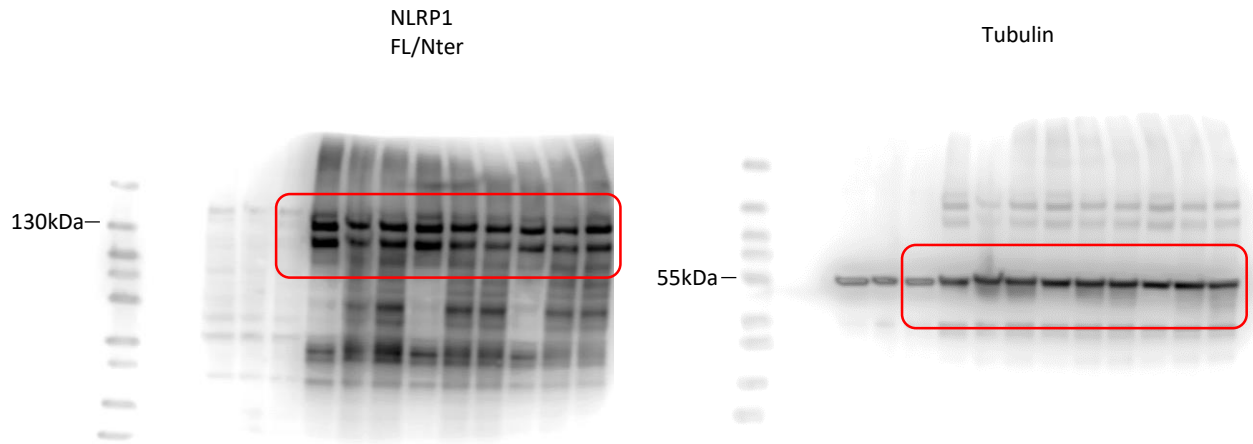

Supplement: SourceData F3 — is the source file for Fig. 3. [file JEM_20230104_SourceDataF3.pdf]

A

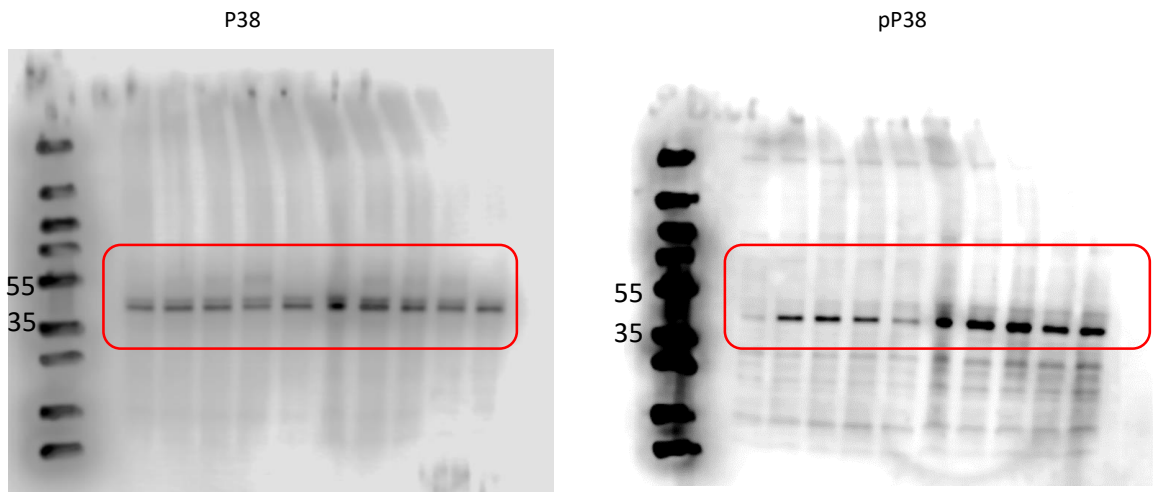

D

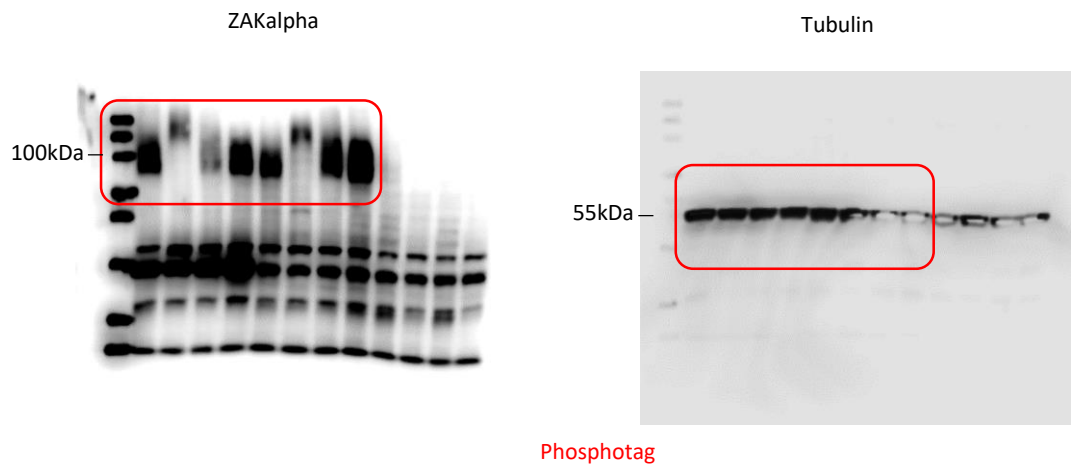

G

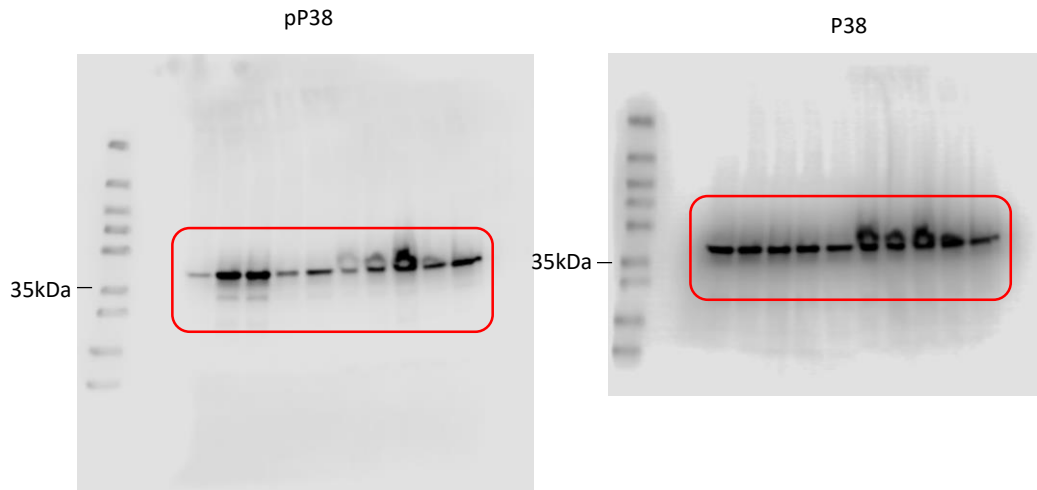

Supplement: SourceData F4 — is the source file for Fig. 4. [file JEM_20230104_SourceDataF4.pdf]

C

NLRP1  
FL/Nter

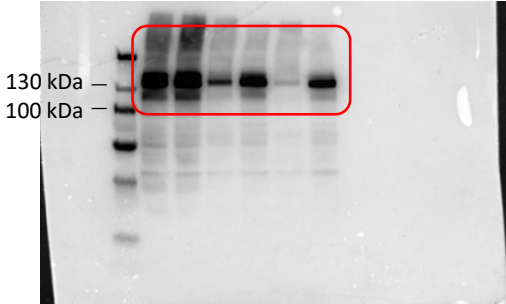

GSDMD (Anti Cter)

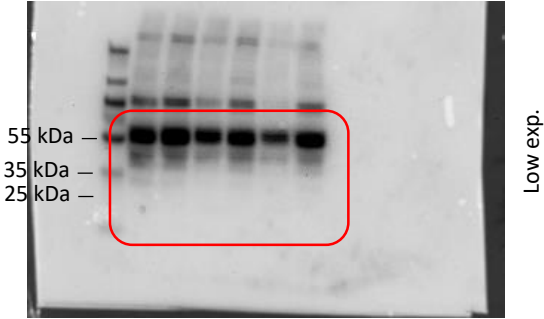

GSDMD (Anti Cter)

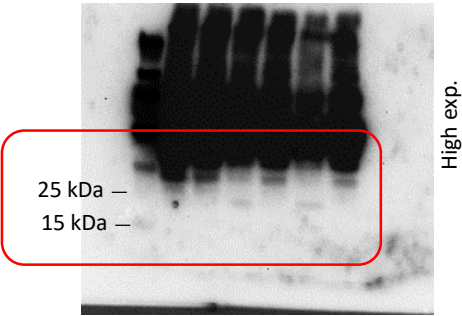

Tubulin

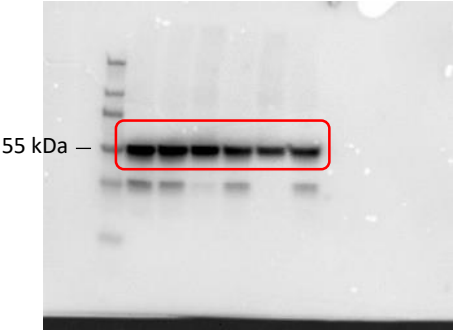

Supplement: SourceData FS1 — is the source file for Fig. S1. [file JEM_20230104_SourceDataFS1.pdf]

# SourceDataFS2

B

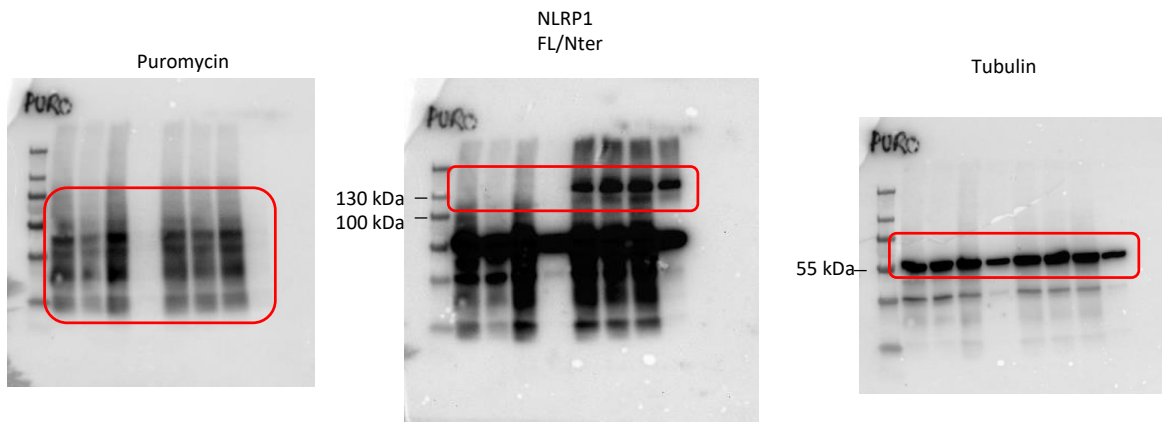

C

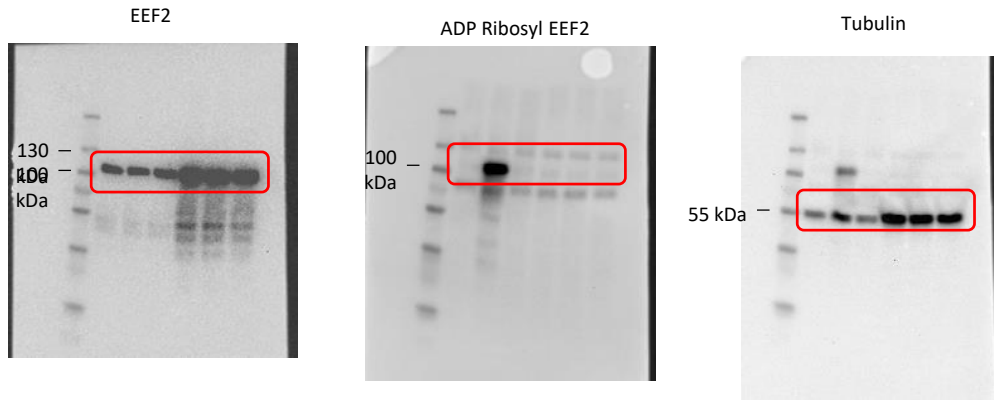

D

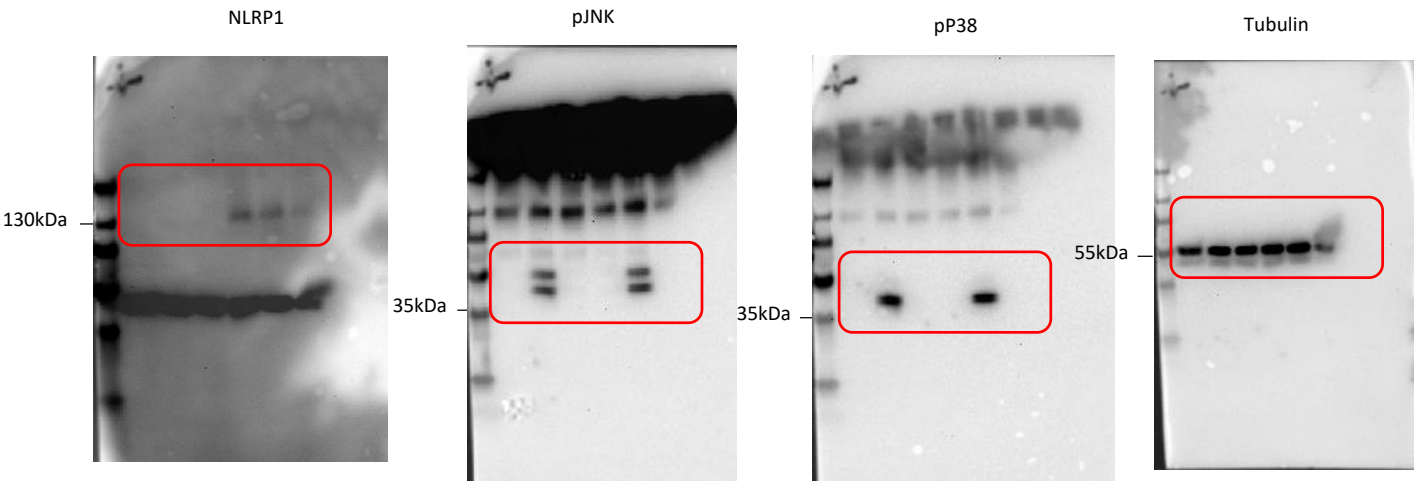

E

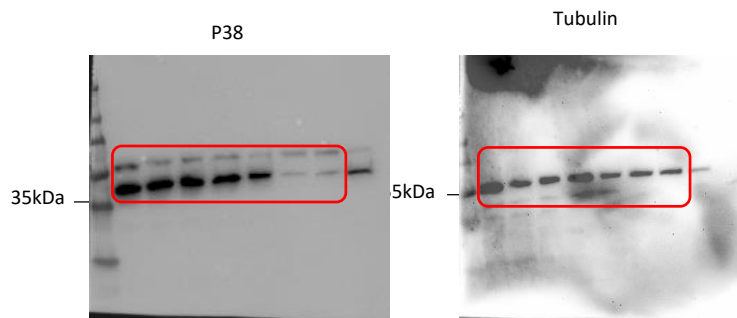

Supplement: SourceData FS2 — is the source file for Fig. S2. [file JEM_20230104_SourceDataFS2.pdf]

SourceDataFS3

**B**

NLRP1

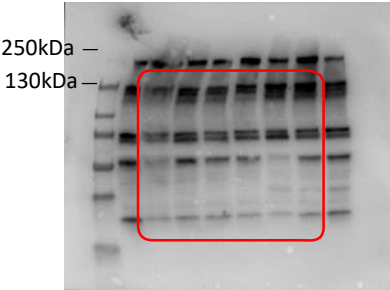

GSDMD

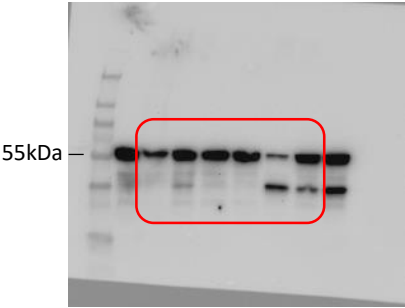

Tubulin

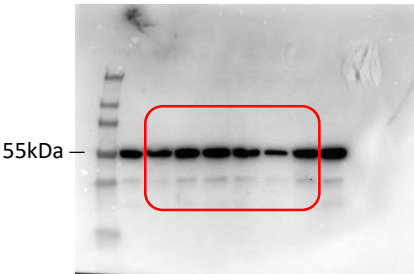

Supplement: SourceData FS3 — is the source file for Fig. S3. [file JEM_20230104_SourceDataFS3.pdf]
